# Enantiodifferentiation of α-Arylacetic Acid and Thiohydantoin Derivatives by NMR Using Thiourea-Based Chiral Solvating Agents

**DOI:** 10.3390/molecules31142526

**Published:** 2026-07-20

**Authors:** Sule Erol Gunal

**Affiliations:** Department of Pharmaceutical Chemistry, Faculty of Pharmacy, İstanbul University-Cerrahpaşa, 34500 Istanbul, Türkiye; sule.gunal@iuc.edu.tr

**Keywords:** chiral solvating agents, enantiodiscrimination, thioureas, NMR spectroscopy, chiral carboxylic acids, thiohydantoins

## Abstract

Two thiourea-based chiral solvating agents (CSAs), ***S-*1** and ***S-*2**, were evaluated for the enantiomeric discrimination of representative α-arylacetic acids by ^1^H NMR spectroscopy in the presence of DMAP. Enantiomeric discrimination was assessed by monitoring chemical shift nonequivalence (ΔΔδ) arising from the formation of diastereomeric host–guest complexes. ***S-*1** exhibited concentration-dependent enantiomeric discrimination toward all investigated carboxylic acid derivatives, reaching a maximum ΔΔδ value of 0.021 ppm. In contrast, ***S-*2** failed to produce detectable signal splitting under identical experimental conditions. A 1D ROESY experiment together with association constant measurements supported the proposed diastereomeric host–guest complexation model and the preferential binding of one enantiomer by ***S-*1**. To further expand the substrate scope, two thiohydantoin derivatives were also examined, and ***S-*1** produced measurable chemical shift nonequivalences, with the largest ΔΔδ value reaching 0.019 ppm. Comparison with previously reported thiourea-based CSAs further highlighted the importance of hydrogen-bonding ability, aromatic anisotropy, and overall molecular architecture in governing enantiomeric discrimination. Overall, these findings provide useful structural insights for the rational design of thiourea-based chiral solvating agents for NMR enantiomeric analysis.

## 1. Introduction

Chirality plays a key role in the biological activity and pharmacological properties of many organic compounds [1]. Chiral molecules exist as non-superimposable mirror images known as enantiomers, which often exhibit significantly different interactions with biological systems [2]. One enantiomer may show the desired therapeutic effect, whereas the other enantiomer may cause undesirable side effects [3,4]. Therefore, separation and quantitative determination of enantiomers is important in pharmaceutical chemistry [5,6,7].

Chiral carboxylic acids are common units in many drug molecules [8,9,10] such as ibuprofen, naproxen, tiagabine and natural products [11,12], where biological activity often depends on their stereochemistry. Therefore, reliable analytical methods for the enantiodiscrimination of chiral carboxylic acids are of great importance. Several analytical techniques have been developed for enantioseparation, including high-performance liquid chromatography (HPLC) [13,14], circular dichroism spectroscopy (CD) [15,16] and gas chromatography (GC) [17] and nuclear magnetic resonance (NMR) spectroscopy [18]. Among these techniques, NMR spectroscopy is a versatile tool because it allows rapid and highly informative analysis without the need for physical separation of enantiomers. However, because enantiomers have the identical NMR spectra in an achiral environment, differentiation between them requires the introduction of a chiral element, such as a chiral derivatizing agent (CDA) [19], chiral solvating agent (CSA) [20] or chiral lanthanide shift reagent [21]. These agents interact with the enantiomers and form diastereomeric complexes with the substrate, resulting in distinguishable NMR signals.

Of the various approaches to NMR enantiodiscrimination, chiral solvating agents have attracted considerable attention due to their ability to enable direct enantiomeric discrimination without chemical derivatization [22]. A wide variety of CSAs, including crown ethers [23], alkaloid derivatives [24,25], phosphoric acids [26], ureas [27], and thioureas [28], have been developed for the recognition of different classes of chiral compounds [29,30,31]. In particular, thiourea-based CSAs have attracted particular attention due to their strong hydrogen-bond donating ability, which promotes the formation of stable and well-defined host–guest complexes with carboxylic acids and other hydrogen-bond-accepting substrates [32,33]. Numerous mono- and bis-thiourea receptors have been reported for the enantiodifferentiation of chiral carboxylic acids by ^1^H NMR spectroscopy, demonstrating that both the hydrogen-bonding motif and the steric environment surrounding the thiourea unit play important roles in molecular recognition [34,35].

**Figure 1:**
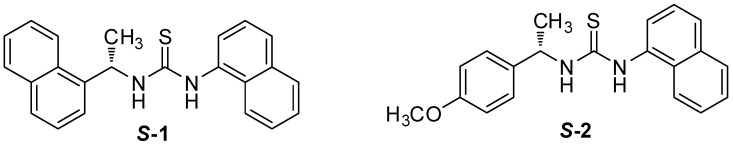
Chiral thioureas (***S-*1** and ***S-*2**) as chiral solvating agents (CSAs).

**Figure 2:**
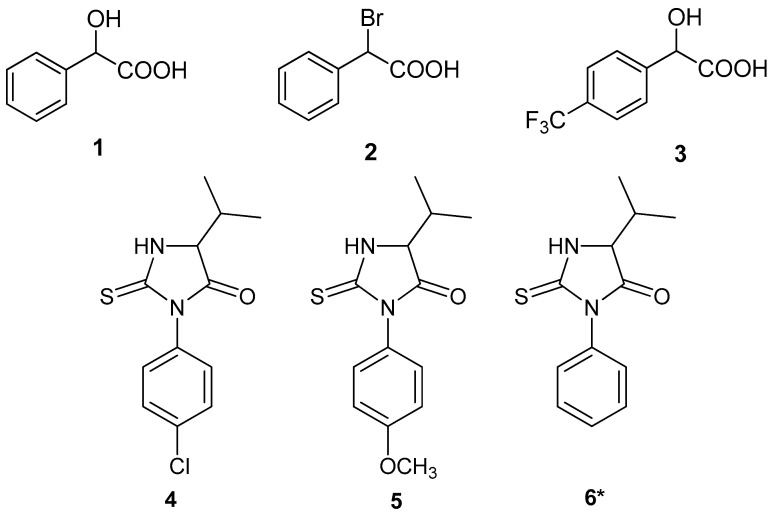
α-Arylacetic acid derivatives (**1**–**3**) and thiohydantoin derivatives (**4**–**6**) investigated for enantiodiscrimination (* Compound **6** was investigated in our previous study [36]).

In the present study, two structurally related chiral thiourea derivatives (***S-*1** and ***S-*2**) shown in Figure 1 were evaluated as CSAs for the enantiodifferentiation of α-arylacetic acid derivatives, namely 2-hydroxy-2-phenylacetic acid (**1**), 2-bromo-2-phenylacetic acid (**2**), and 2-hydroxy-2-(4-(trifluoromethyl)phenyl)acetic acid (**3**). Enantiodifferentiation was investigated by ^1^H NMR spectroscopy through analysis of chemical shift differences (ΔΔδ) induced upon complex formation with the CSAs. The effect of a structural modification between the two thiourea-based CSAs—namely, the replacement of a naphthyl group in ***S-*1** with a 4-methoxyphenyl group in ***S-*2**—on enantiodiscrimination performance and molecular recognition was examined. To broaden the substrate scope, selected thiohydantoin derivatives (**4** and **5**) shown in Figure 2 were also evaluated for their enantiodiscrimination behavior.

Accordingly, the aim of this study was to assess the potential of structurally related chiral thiourea-based CSAs for NMR-based enantiodiscrimination and to gain insight into the influence of structural modification on molecular recognition.

## 2. Results and Discussion

Chiral thioureas (***S-*1** and ***S-*2**) were synthesized via the reaction of (*S*)-1-naphthylethylamine or (*S*)-4-methoxyphenylethylamine with 1-naphthyl isothiocyanate. In our previous study [37], thiourea-based CSAs require 4-dimethylaminopyridine (DMAP) for effective enantiodiscrimination of carboxylic acids, as no significant discrimination is observed in its absence. This behavior is consistent with earlier literature reports [34,35], which emphasize the crucial role of DMAP in facilitating efficient host–guest complex formation and enhancing enantiomeric differentiation. This enhancement is attributed to the formation of a carboxylate–DMAPH^+^ ion pair, which strengthens hydrogen-bonding interactions with the CSA and promotes the formation of diastereomeric complexes. Therefore, in the present study, enantiodiscrimination of α-arylacetic acids was conducted in the presence of 1 equivalent of DMAP.

First, the enantiodiscrimination ability of the thiourea ***S-*1** was evaluated for of α-arylacetic acids (**1**–**3**) by monitoring chemical shift differences (∆∆δ) of α-H signals in the ^1^H NMR spectra at different equivalents of CSA (Table 1).

***S-*1** showed concentration-dependent discrimination for all three substrates, with ΔΔδ values increasing with higher CSA equivalents. For the carboxylic acid derivative **1**, no peak separation was observed at 1–3 equivalents. However, starting from 4 equivalents, a signal splitting appeared, with ∆∆δ increasing from 0.007 ppm (4 equivalents) to 0.011 ppm (6 equivalents) (Table 1).

**Figure 3:**
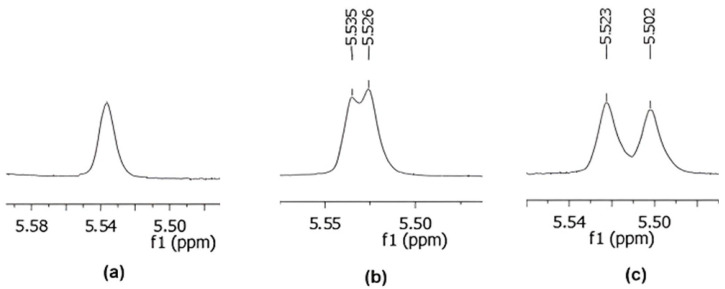
Partial ^1^H NMR spectra showing the α-H of **2** (**a**) in the absence of ***S-*1**, (**b**) in the presence of 3 equivalents of ***S-*1** and (**c**) in the presence of 6 equivalents of ***S-*1**.

For substrates **2** and **3**, measurements were directly initiated at 3 equivalents of ***S-*1**, since 1 and 2 equivalents were found to be insufficient for effective enantiodifferentiation based on experiment with substrate 1. Consistent with this expectation, substrate **2** exhibited detectable enantiomeric differentiation at 3 equivalents (ΔΔδ = 0.009 ppm), and the signal separation increased progressively with increasing amounts of ***S-*1**, reaching 0.021 ppm at 6 equivalents (Figure 3). Among the investigated α-arylacetic acids, substrate **2** showed the largest chemical shift differences (Table 1).

For the substrate **3**, discrimination was weaker. No splitting was observed at 3 and 4 equivalents, while small ∆∆δ values were observed at higher equivalents (0.004 ppm and 0.008 ppm at 5 and 6 equivalents, respectively).

The differences in enantiodiscrimination observed among substrates **1**–**3** are likely influenced by a combination of electronic and steric effects arising from the α-substituent and the aromatic ring substitution pattern. Substrate **2**, bearing a Br atom at the α-position, exhibits the largest ΔΔδ value. The electron withdrawing inductive effect of Br may increase the acidity of the carboxylic acid, favoring the formation of the carboxylate–DMAPH^+^ ion pair and strengthening the hydrogen-bonding interactions with ***S-*1**. In addition, the α-Br substituent may provide a more favorable spatial arrangement within the chiral binding environment. In contrast, substrates **1** and **3** both contain an α-hydroxyl group, which can participate in additional intra- or intermolecular hydrogen-bonding interactions. These interactions may alter the preferred binding geometry and reduce the magnetic differentiation between the resulting diastereomeric complexes. Furthermore, the para-CF_3_ substituent in substrate **3** further modifies the electronic properties of the aromatic ring and may influence the overall orientation of the substrate within the host–guest complex. Overall, the results indicate that both the nature of the α-substituent and the electronic characteristics of the aromatic ring contribute to the efficiency of enantiodiscrimination.

**Figure 4:**
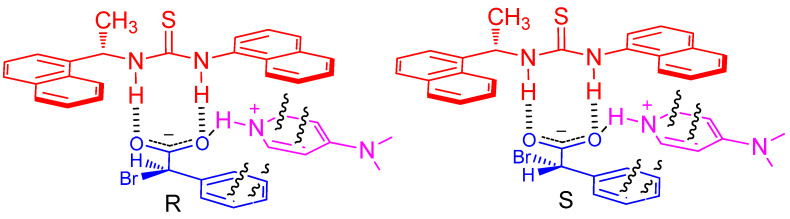
The hypothetical model for diastereomeric complexes formed between R and S enantiomers of **2** and thiourea ***S-*1** in the presence of DMAP.

The enantiodiscrimination observed for ***S-*1** is likely associated with its structural features. The presence of two naphthyl groups generates a rigid and extended π-system. This structural arrangement may facilitate favorable aromatic interactions with the aromatic part of the substrates, thus promoting stable complex formation (Figure 4). In addition, the rigid framework may contribute to the formation of a well-defined chiral pocket around the thiourea functionality, which enhances stereochemical differentiation. Furthermore, DMAP and the enantiomers of a racemic α-arylacetic acid associate through the formation of a carboxylate–DMAPH^+^ ion pair via an NH···O hydrogen bond (Figure 4). This interaction may also strengthen intermolecular hydrogen bonding between ***S-*1** and carboxylic acid. These combined interactions stabilize diastereomeric complexes in which the α-hydrogen atoms of the two enantiomers of the α-arylacetic acid experience different chemical environments, thus leading to observable chemical shift differences (Figure 4).

To provide experimental support for the proposed host–guest interaction, a 1D ROESY experiment was performed for ***S-*1** and substrate **2** (Figure S31). Upon selective irradiation, ROESY correlations were observed between the α-H of 2 and the methyl (CH_3_) and methine (CH) protons of ***S-*1**. Additional intermolecular correlations were also detected between the substrate α-H and several aromatic protons of ***S-*1**. These correlations indicate that the α-carbon region of the substrate is positioned in close spatial proximity to the chiral center and the aromatic framework of the CSA. Although the 1D ROESY experiment does not define the exact binding geometry, it supports the formation of the diastereomeric complex responsible for the observed enantiodiscrimination.

**Figure 5:**
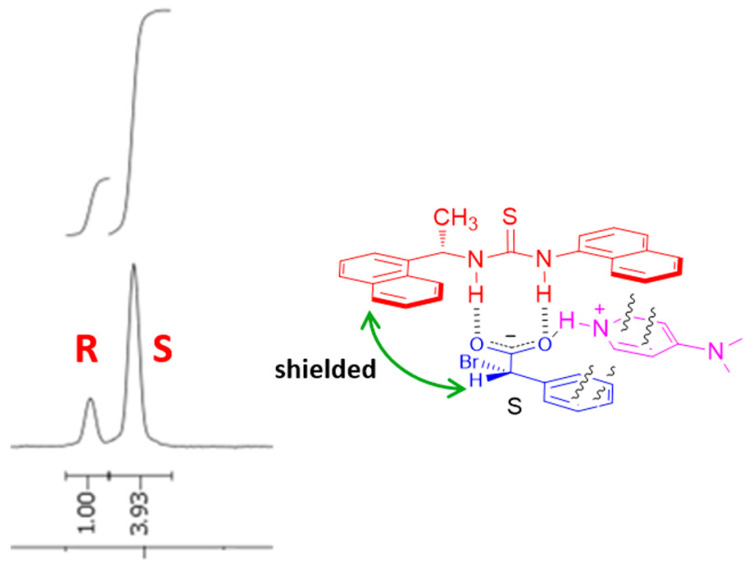
^1^H NMR signals showing α-H of substrate **2** (the ratio of (R)-2:(S)-2 is 1:4) and the proposed diastereomeric complexes formed between ***S-*1** and S enantiomer of **2** in the presence of DMAP.

The proposed complexation model suggests that the (S)-enantiomer may experience a stronger shielding effect because its α-H is positioned within the shielding region of the naphthyl ring of ***S-*1**. To examine this possibility, the ^1^H NMR spectrum of a 1:4 mixture of **(R)-2**:**(S)-2** was recorded. The α-H signal of the *S-*enantiomer of **2** appeared at a more shielded position than that of the R-enantiomer, indicating a stronger shielding effect (Figure 5). This chemical shift difference supports the preferential positioning of the *S-*enantiomer within the aromatic environment of ***S-*1**.

The association constants (Ka) of the complexes formed between ***S-*1** and the enantiomers of substrate **2** were determined to evaluate the relative binding strengths. The Ka value for the R-enantiomer was calculated to be 13.10 M^−1^, while the *S-*enantiomer showed a higher value of 28.73 M^−1^ (Figure S32). The stronger binding observed for the *S-*enantiomer indicates a more favorable interaction with ***S-*1**, which may result from improved hydrogen-bonding interactions and steric compatibility between the substrate and the thiourea–naphthyl framework. The difference in association constants provides quantitative evidence for the preferential recognition of the *S-*enantiomer by ***S-*1**.

**Figure 6:**
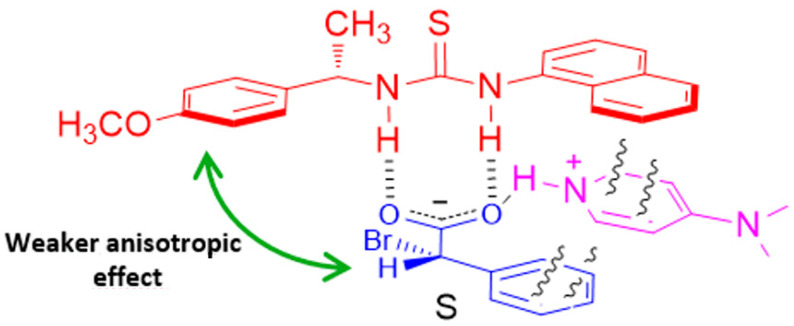
The hypothetical model for diastereomeric complexes formed between S enantiomer of **2** and thiourea ***S-*2** in the presence of DMAP.

To further understand the structural factors governing enantioselective recognition, the enantiodiscrimination ability of ***S-*2** was also examined. Although ΔΔδ was observed for thiourea ***S-*1**, no ΔΔδ was observed for thiourea ***S-*2** with any of the tested α-arylacetic acids, even at higher equivalents. All signals remained as single peaks, with no measurable ΔΔδ values. Structurally, ***S-*2** differs from ***S-*1** in that one of the chiral naphthylethyl groups is replaced by a chiral 4-methoxyphenylethyl group. This structural modification replaces the strongly anisotropic naphthyl ring with the comparatively weaker anisotropic effect of a phenyl ring (Figure 6). As a consequence, the spatial shielding/deshielding environment experienced by substrate is expected to be less pronounced. In addition, the electron-donating methoxy substituent can modulate the electronic properties of the aromatic ring and may influence hydrogen bond donating ability of thiourea NH groups. These combined effects are expected to reduce the strength and the geometrical rigidity of host–guest interactions. As a result, ***S-*2** is expected to form less differentiated diastereomeric complexes with the substrates, leading to reduced enantiodiscrimination compared to ***S-*1**.

**Figure 7:**
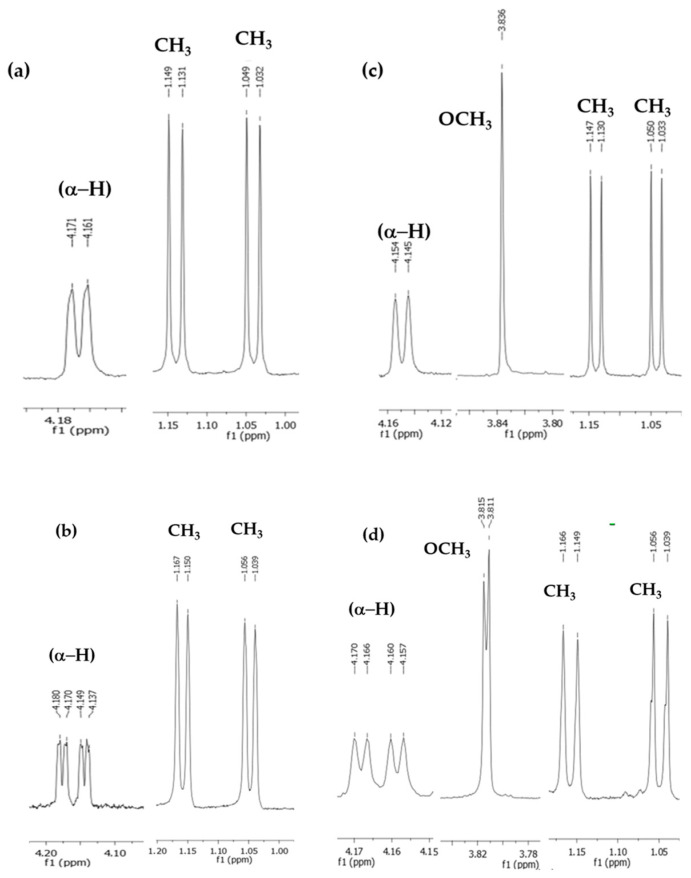
Partial ^1^H NMR spectra of **4** and **5** recorded in CDCl**_3_**. (**a**) α-H and diastereotopic CH_3_ signals of **4** in the absence of ***S-*1**. (**b**) α-H and diastereotopic CH_3_ signals of **4** in the presence of 6 equivalents of ***S-*1**. (**c**) α-H, OCH_3_, and diastereotopic CH_3_ signals of **5** in the absence of ***S-*1**. (**d**) α-H, OCH_3_, and diastereotopic CH_3_ signals of **5** in the presence of 6 equivalents of ***S-*****1**.

To broaden the substrate scope, thiohydantoin derivatives **4** and **5** shown in Figure 2 were also examined as additional substrates in the presence of ***S-*1**. As summarized in Table 2 and shown in Figure 7, both substrates exhibit only small but measurable chemical shift differences upon complexation. For substrate **4**, the α-H proton shows the largest shift difference (ΔΔδ = 0.019 ppm), suggesting that this position is most strongly influenced by the anisotropic shielding environment of the naphthyl framework of ***S-*1**. In comparison, substrate **5** displays a reduced α-H shift difference (ΔΔδ = 0.011 ppm). The methoxy substituent in **5** gives rise to only a very small shift difference (ΔΔδ = 0.004 ppm), whereas the isopropyl methyl groups in both substrates remain unaffected, indicating that these peripheral aliphatic groups do not participate significantly in the recognition process.

**Figure 8:**
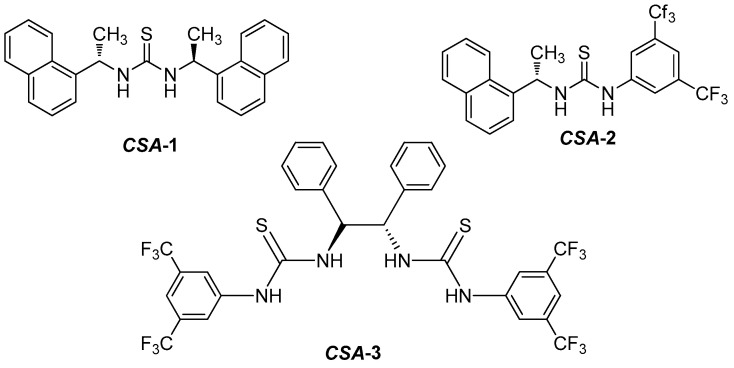
Previously reported thioureas as CSAs [34,37].

In our previous study [37], C2-symmetrical thiourea bearing two chiral naphthylethyl units (**CSA-1**) shown in Figure 8 exhibited superior enantiodiscrimination abilities toward chiral carboxylic acids, producing ΔΔδ values of 0.130–0.167 ppm for the same α-arylacetic acid derivatives (Table 3). In the present work, however, ***S-*1**, bearing a single chiral naphthylethyl unit, afforded considerably smaller ΔΔδ values, with the maximum ΔΔδ reaching only 0.021 ppm for the α-H proton of **2**, despite the use of the same substrates and DMAP-assisted conditions. Since both studies rely on the formation of diastereomeric thiourea–carboxylate–DMAPH^+^ complexes, the observed differences in discrimination efficiency must originate primarily from the structural features of the CSA. **CSA-1** was also previously reported to exhibit enantiodifferentiation for thiohydantoin **6**, with ΔΔδ values of 0.057 ppm (α-H) and 0.017 ppm (CH_3_) [36] (Table 3).

The higher enantiodiscrimination ability of the **CSA-1** cannot be attributed solely to C_2_ symmetry. Because the unsymmetrical thiourea **CSA-2** (Figure 8), which combines a chiral naphthylethyl unit with a 3,5-bis(trifluoromethyl)phenyl group, also proved highly effective, affording ΔΔδ values of up to 0.187 ppm (Table 3). The strongly electron-withdrawing CF_3_ substituents are expected to increase the acidity of the thiourea NH protons, thereby strengthening the hydrogen bonding interactions with the carboxylate–DMAPH^+^ ion pair. Simultaneously, the naphthyl group provides an extended anisotropic aromatic surface capable of generating distinct magnetic environments for the two diastereomeric complexes. In contrast, the thioureas investigated in the present study, ***S-*1** and ***S-*2**, although containing naphthyl units, lack strongly electron-withdrawing substituents and therefore likely form less differentiated host–guest complexes.

Compared with the bisthiourea CSA reported by Bian et al. (**CSA-3**) (Figure 8) [34], which afforded ΔΔδ values of 0.204, 0.127, and 0.177 ppm for substrates **1**–**3**, respectively (Table 3), the superior enantiodiscrimination performance of **CSA-3** can be attributed to its C2-symmetric bisthiourea architecture, which provides two cooperative thiourea hydrogen-bonding sites capable of simultaneously interacting with the substrate. Moreover, the presence of electron-withdrawing 3,5-bis(trifluoromethyl)phenyl substituents enhances the acidity of the thiourea NH protons, resulting in stronger hydrogen-bond donor ability and the formation of more stable diastereomeric host–guest complexes. In contrast, ***S-*1** contains a single thiourea unit linked to a naphthyl-substituted chiral scaffold, which offers fewer cooperative binding interactions and lower NH acidity. Consequently, the weaker host–guest interactions lead to reduced enantiomeric discrimination, as reflected by the smaller ΔΔδ values. These results highlight that cooperative hydrogen bonding and increased NH acidity are critical structural features governing the enantiodiscrimination efficiency of thiourea-based chiral solvating agents.

## 3. Materials and Methods

### 3.1. General

All reagents and solvents were obtained commercially (Sigma-Aldrich, St. Louis, MO, USA; Merck, Darmstadt, Germany; TCI, Tokyo, Japan) and used without further purification. Melting points were determined on Electrothermal 9100 melting point apparatus. ^1^H and ^13^C{^1^H} NMR measurements were carried out in CDCl_3_ solution on a Varian Mercury VX-400 spectrometer (Palo Alto, CA, USA) operating at 400 MHz and 100 MHz for the ^1^H and ^13^C nuclei, respectively. The temperature was maintained at 298 ± 0.1 K. The ^1^H NMR spectra were acquired using 32 scans. The 1D ROESY spectra were recorded using a selective inversion pulse with 512 scans, a relaxation delay of 5 s, and a mixing time of 0.3 s. The NMR spectra were processed using MestReNova version 6.0.2-5475 (Mestrelab Research S.L., Santiago de Compostela, Spain). The reproducibility of the NMR measurements was evaluated on selected representative samples, and the results were found to be consistent.

### 3.2. Synthesis of CSAs

***S-*1** and ***S-*2** were synthesized by the reaction of 1-naphthyl isothiocyanate (5 mmol) with (*S*)-1-naphthylethylamine (5 mmol) or (*S*)-4-methoxyphenylethylamine (5 mmol) in dichloromethane at room temperature (10 mL) for 3 h.

#### 3.2.1. (*S*)-1-(Naphthalen-1-yl)-3-(1-(naphthalen-1-yl)ethyl)thiourea (***S-*1**)

White solid, yield:1.39 g (78%), mp: 126–127 ^°^C. ^1^H NMR (400 MHz, CDCl_3_) δ 8.27 (d, *J* = 8.4 Hz, 1H, ArH), 8.13 (br, 1H, NH) 7.94–7.86 (m, 5H, ArH), 7.68–7.05 (m, 8H, ArH), 6.43 (br, 1H, CH), 5.87 (s, 1H, NH), 1.67 (d, *J* = 6.6 Hz, 3H, CH_3_). ^13^C NMR (100 MHz, CDCl_3_) δ 180.36, 137.31, 134.56, 133.87, 131.55, 131.25, 129.67, 128.88, 128.79, 128.71, 128.58, 128.39, 127.27, 127.03, 126.77, 125.95, 125.71, 124.98, 123.89, 122.84, 122.61, 51.08, 20.02. Calculated for C_23_H_20_N_2_S: C, 77.49; H, 5.65; N, 7.86. Found: C, 77.39; H, 5.67; N, 7.99.

#### 3.2.2. (*S*)-1-(1-(4-Methoxyphenyl)ethyl)-3-(naphthalen-1-yl)thiourea (***S-*2**)

White solid, yield: 1.24 g (74%), mp: 181–182 ^°^C. ^1^H NMR (400 MHz, CDCl_3_) δ 7.97 (br, 1H, NH) 7.93–7.44 (m, 3H, ArH), 7.58–7.43 (m, 4H, ArH),7.14–6.80 (m, 4H, ArH), 5.87 (br, 1H, CH), 5.67 (s, 1H, NH), 3.78 (s, 3H, OCH_3_), 1.44 (d, *J* = 6.4 Hz, 3H, CH_3_). ^13^C NMR (100 MHz, CDCl_3_) δ 180.65, 158.86, 134.67, 134.25, 131.86, 131.64, 129.82, 128.98, 128.53, 127.44, 127.41, 127.37, 127.13, 125.75, 125.10, 122.57, 113.95, 55.29, 53.80, 21.22. Calculated for C_20_H_20_N_2_OS: C, 71.40; H, 5.99; N, 8.33. Found: C, 71.52; H, 5.89; N, 8.39.

### 3.3. Sample Preparation for NMR Analysis

Carboxylic acid (3 μmol, 5.0 mM, 1 equiv), DMAP (3 μmol, 5.0 mM, 1 equiv), and varying equivalents of the chiral solvating agent were dissolved in CDCl_3_ (0.6 mL) directly in an NMR tube. ^1^H NMR spectra were recorded on a 400 MHz spectrometer.

## 4. Conclusions

In this study, two chiral thiourea-based CSAs, ***S-*1** and ***S-*2**, were synthesized and evaluated for the enantiodiscrimination of representative chiral carboxylic acids and thiohydantoin derivatives by ^1^H NMR spectroscopy. ***S-*1** exhibited measurable enantiomeric discrimination, whereas ***S-*2** failed to generate detectable ΔΔδ values under identical experimental conditions. The better performance of ***S-*1** is likely related to the presence of the naphthyl group, which provides a larger anisotropic aromatic surface and may promote more favorable host–guest interactions, including aromatic interactions, resulting in a more distinct magnetic environment for the diastereomeric complexes. In contrast, replacement of one naphthyl group with a 4-methoxyphenyl substituent in ***S-*2** reduces both the aromatic anisotropy and the effectiveness of host–guest interactions, resulting in negligible enantiodifferentiation.

Although ***S-*1** afforded measurable enantiomeric discrimination for the investigated carboxylic acid and thiohydantoin derivatives, the observed ΔΔδ values were generally modest compared with those reported for previously developed thiourea-based chiral solvating agents. These results emphasize the critical role of CSA structure in determining enantiodiscrimination efficiency and suggest that further structural optimization, together with evaluation using a broader range of substrates, may contribute to the development of more effective thiourea-based chiral solvating agents.

## Figures and Tables

**Table 1:** ^1^H NMR chemical shifts (δ, ppm) and the chemical shift differences (ΔΔδ, ppm) of α-H of racemic carboxylic acids at varying equivalents (eq) of CSAs in CDCl_3_.

CSA	Carboxylic Acid	Eq.	δ (ppm)	∆∆δ (ppm)
***S-*** **1**	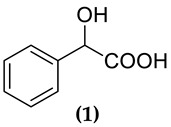	1	5.050	-
		2	5.041	-
		3	5.035	-
		4	5.033 and 5.026	0.007
		5	5.021 and 5.013	0.008
		6	5.020 and 5.009	0.011
	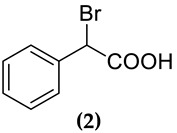	3	5.535 and 5.526	0.009
		4	5.533 and 5.520	0.013
		5	5.530 and 5.513	0.017
		6	5.523 and 5.502	0.021
	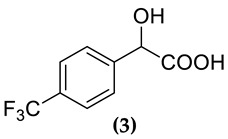	3	5.088	-
		4	5.080	-
		5	5.072 and 5.068	0.004
		6	5.069 and 5.061	0.008
***S-*** **2**	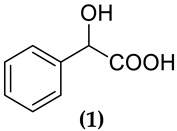	3	5.048	-
		4	5.046	-
		5	5.045	-
		6	5.044	-
	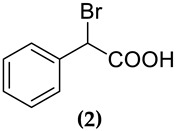	3	5.536	-
		4	5.533	-
		5	5.527	-
		6	5.524	-
	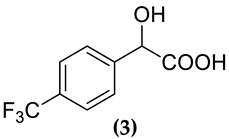	3	5.089	-
		4	5.082	-
		5	5.079	-
		6	5.077	-

**Table 2:** Chemical shift differences (ΔΔδ, ppm) of racemic 5-Isopropyl-2-thiohydantoins (**4–5**) in the presence of 6 equivalents of ***S-*1**.

Substrate	Proton	∆∆δ (ppm)
**4**	α-H	0.019
	CH_3_	-
**5**	α-H	0.011
	OCH_3_	0.004
	CH_3_	-

**Table 3:** ^1^H NMR chemical shift differences (ΔΔδ, ppm) of selected substrates enantiodiscriminated by previously reported thioureas.

CSA	Substrate	Proton	∆∆δ (ppm)
**CSA-1** ^a^	**1**	α-H	0.130
	**2**	α-H	0.167
	**3**	α-H	0.127
	**6**	α-H	0.057
		CH_3_	0.017
**CSA-2** ^a^	**1**	α-H	0.090
	**2**	α-H	0.187
	**3**	α-H	0.084
**CSA-3** ^b^	**1**	α-H	0.204
	**2**	α-H	0.127
	**3**	α-H	0.177

^a^ See refs. [36,37]; ^b^ See ref. [34].

## Data Availability

Data is contained within the article or Supplementary Materials.

## Supplementary Materials

The following supporting information can be downloaded at: https://www.mdpi.com/article/10.3390/molecules31142526/s1, Figure S1: ^1^H NMR spectrum of ***S-*1** in CDCl_3_; Figure S2: ^13^C NMR spectrum of ***S-*1** in CDCl_3_; Figure S3: ^1^H NMR spectrum 1 in the presence of 1 equivalent of ***S-*1** and DMAP in CDCl_3_; Figure S4: ^1^H NMR spectrum of 1 in the presence of 2 equivalents of ***S-*1** and DMAP in CDCl_3_; Figure S5: ^1^H NMR spectrum of 1 in the presence of 3 equivalents of ***S-*1** and DMAP in CDCl_3_; Figure S6: ^1^H NMR spectrum of 1 in the presence of 4 equivalents of ***S-*1** and DMAP in CDCl_3_; Figure S7: ^1^H NMR spectrum of 1 in the presence of 5 equivalents of ***S-*1** and DMAP in CDCl_3_; Figure S8: ^1^H NMR spectrum of 1 in the presence of 6 equivalents of ***S-*1** and DMAP in CDCl_3_; Figure S9: ^1^H NMR spectrum of 2 in the presence of 3 equivalents of ***S-*1** and DMAP in CDCl_3_; Figure S10: ^1^H NMR spectrum of 2 in the presence of 4 equivalents of ***S-*1** and DMAP in CDCl_3_; Figure S11: ^1^H NMR spectrum of 2 in the presence of 5 equivalents of ***S-*1** and DMAP in CDCl_3_; Figure S12: ^1^H NMR spectrum of 2 in the presence of 6 equivalents of ***S-*1** and DMAP in CDCl_3_; Figure S13: ^1^H NMR spectrum of 3 in the presence of 3 equivalents of ***S-*1** and DMAP in CDCl_3_; Figure S14: ^1^H NMR spectrum of 3 in the presence of 4 equivalents of ***S-*1** and DMAP in CDCl_3_; Figure S15: ^1^H NMR spectrum of 3 in the presence of 5 equivalents of ***S-*1** and DMAP in CDCl_3_; Figure S16: ^1^H NMR spectrum of 3 in the presence of 6 equivalents of ***S-*1** and DMAP in CDCl_3_; Figure S17: ^1^H NMR spectrum of ***S-*2** in CDCl_3_; Figure S18: ^13^C NMR spectrum of ***S-*2** in CDCl_3_; Figure S19: ^1^H NMR spectrum of 1 in the presence of 3 equivalents of ***S-*2** and DMAP in CDCl_3_; Figure S20: ^1^H NMR spectrum of 1 in the presence of 4 equivalents of ***S-*2** and DMAP in CDCl_3_; Figure S21: ^1^H NMR spectrum of 1 in the presence of 5 equivalents of ***S-*2** and DMAP in CDCl_3_; Figure S22: ^1^H NMR spectrum of 1 in the presence of 6 equivalents of ***S-*2** and DMAP in CDCl_3_; Figure S23: ^1^H NMR spectrum of 2 in the presence of 3 equivalents of ***S-*2** and DMAP in CDCl_3_; Figure S24: ^1^H NMR spectrum of 2 in the presence of 4 equivalents of ***S-*2** and DMAP in CDCl_3_; Figure S25: ^1^H NMR spectrum of 2 in the presence of 5 equivalents of ***S-*2** and DMAP in CDCl_3_; Figure S26: ^1^H NMR spectrum of 2 in the presence of 6 equivalents of ***S-*2** and DMAP in CDCl_3_; Figure S27: ^1^H NMR spectrum of 3 in the presence of 3 equivalents of ***S-*2** and DMAP in CDCl_3_; Figure S28: ^1^H NMR spectrum of 3 in the presence of 4 equivalents of ***S-*2** and DMAP in CDCl_3_; Figure S29: ^1^H NMR spectrum of 3 in the presence of 5 equivalents of ***S-*2** and DMAP in CDCl_3_; Figure S30: ^1^H NMR spectrum of 3 in the presence of 6 equivalents of ***S-*2** and DMAP in CDCl_3_; Figure S31: 1D-ROESY (400 MHz, CDCl_3_, 298 K, mixing time 500 ms) spectrum of complex formed between ***S-*1** and substrate 2; Figure S32: Association constants calculated for (a) R enantiomer and (b) S enantiomer of 2 with ***S-*1** in the presence of DMAP; Figure S33: ^1^H NMR spectrum of 4 in the presence of 6 equivalents of ***S-*1** in CDCl_3_; Figure S34: ^1^H NMR spectrum of 5 in the presence of 6 equivalents of ***S-*1** in CDCl_3_.
