# Supplementary material for: Enantiodifferentiation of α-Arylacetic Acid and Thiohydantoin Derivatives by NMR Using Thiourea-Based Chiral Solvating Agents
Source: Molecules. 2026 Jul 20;31(14):2526. doi: 10.3390/molecules31142526 (PMC13416210; doi:10.3390/molecules31142526)
Supplement: Supplementary file 1 [file molecules-31-02526-s001.zip › molecules-4391070-supplementary.pdf]

Supporting Information

Enantiodifferentiation of α-Arylacetic Acid and Thiohydantoin Derivatives by NMR Using Thiourea-Based Chiral Solvating Agents

Sule Erol Gunal*

Department of Pharmaceutical Chemistry, Faculty of Pharmacy, İstanbul University-Cerrahpaşa, 34500 Istanbul, Türkiye; sule.gunal@iuc.edu.tr

Table of Contents...S1-S2
Figure S1. ^{1}H NMR spectrum of ***S*-1** in $CDCl_3$..S3
Figure S2. ^{13}C NMR spectrum of ***S*-1** in $CDCl_3$..S3
Figure S3. ^{1}H NMR spectrum **1** in the presence of 1 equivalent of ***S*-1** and DMAP in $CDCl_3$..S4
Figure S4. ^{1}H NMR spectrum of **1** in the presence of 2 equivalents of ***S*-1** and DMAP in $CDCl_3$..S4
Figure S5. ^{1}H NMR spectrum of **1** in the presence of 3 equivalents of ***S*-1** and DMAP in $CDCl_3$..S5
Figure S6. ^{1}H NMR spectrum of **1** in the presence of 4 equivalents of ***S*-1** and DMAP in $CDCl_3$..S5
Figure S7. ^{1}H NMR spectrum of **1** in the presence of 5 equivalents of ***S*-1** and DMAP in $CDCl_3$..S6
Figure S8. ^{1}H NMR spectrum of **1** in the presence of 6 equivalents of ***S*-1** and DMAP in $CDCl_3$..S6
Figure S9. ^{1}H NMR spectrum of **2** in the presence of 3 equivalents of ***S*-1** and DMAP in $CDCl_3$..S7
Figure S10. ^{1}H NMR spectrum of **2** in the presence of 4 equivalents of ***S*-1** and DMAP in $CDCl_3$..S7
Figure S11. ^{1}H NMR spectrum of **2** in the presence of 5 equivalents of ***S*-1** and DMAP in $CDCl_3$..S8
Figure S12. ^{1}H NMR spectrum of **2** in the presence of 6 equivalents of ***S*-1** and DMAP in $CDCl_3$..S8
Figure S13. ^{1}H NMR spectrum of **3** in the presence of 3 equivalents of ***S*-1** and DMAP in $CDCl_3$..S9
Figure S14. ^{1}H NMR spectrum of **3** in the presence of 4 equivalents of ***S*-1** and DMAP in $CDCl_3$..S9
Figure S15. ^{1}H NMR spectrum of **3** in the presence of 5 equivalents of ***S*-1** and DMAP in $CDCl_3$..S10
Figure S16. ^{1}H NMR spectrum of **3** in the presence of 6 equivalents of ***S*-1** and DMAP in $CDCl_3$..S10
Figure S17. ^{1}H NMR spectrum of ***S*-2** in $CDCl_3$..S11
Figure S18. ^{13}C NMR spectrum of ***S*-2** in $CDCl_3$..S11
Figure S19. ^{1}H NMR spectrum of **1** in the presence of 3 equivalents of ***S*-2** and DMAP in $CDCl_3$..S12

Figure S20. ^{1}H NMR spectrum of **1** in the presence of 4 equivalents of ***S*-2** and DMAP in $CDCl_3$……………………………………………………………………**S12**
Figure S21. ^{1}H NMR spectrum of **1** in the presence of 5 equivalents of ***S*-2** and DMAP in $CDCl_3$……………………………………………………………………**S13**
Figure S22. ^{1}H NMR spectrum of **1** in the presence of 6 equivalents of ***S*-2** and DMAP in $CDCl_3$……………………………………………………………………**S13**
Figure S23. ^{1}H NMR spectrum of **2** in the presence of 3 equivalents of ***S*-2** and DMAP in $CDCl_3$……………………………………………………………………**S14**
Figure S24. ^{1}H NMR spectrum of **2** in the presence of 4 equivalents of ***S*-2** and DMAP in $CDCl_3$……………………………………………………………………**S14**
Figure S25. ^{1}H NMR spectrum of **2** in the presence of 5 equivalents of ***S*-2** and DMAP in $CDCl_3$……………………………………………………………………**S15**
Figure S26. ^{1}H NMR spectrum of **2** in the presence of 6 equivalents of ***S*-2** and DMAP in $CDCl_3$……………………………………………………………………**S15**
Figure S27. ^{1}H NMR spectrum of **3** in the presence of 3 equivalents of ***S*-2** and DMAP in $CDCl_3$……………………………………………………………………**S16**
Figure S28. ^{1}H NMR spectrum of **3** in the presence of 4 equivalents of ***S*-2** and DMAP in $CDCl_3$……………………………………………………………………**S16**
Figure S29. ^{1}H NMR spectrum of **3** in the presence of 5 equivalents of ***S*-2** and DMAP in $CDCl_3$……………………………………………………………………**S17**
Figure S30. ^{1}H NMR spectrum of **3** in the presence of 6 equivalents of ***S*-2** and DMAP in $CDCl_3$……………………………………………………………………**S17**
Figure S31. 1D-ROESY (400 MHz, $CDCl_3$, 298 K, mixing time 500 ms) spectrum of complex formed between S-1 and substrate 2……………………………………………… **S18**
Figure S32. Association constants calculated for a) R enantiomer and b) S enantiomer of 2 with S-1 in the presence of DMAP. ……………………………………………… **S18**
Figure S33. ^{1}H NMR spectrum of **4** in the presence of 6 equivalents of ***S*-1** in $CDCl_3$……..**S19**
Figure S34. ^{1}H NMR spectrum of **5** in the presence of 6 equivalents of ***S*-1** in $CDCl_3$……..**S19**

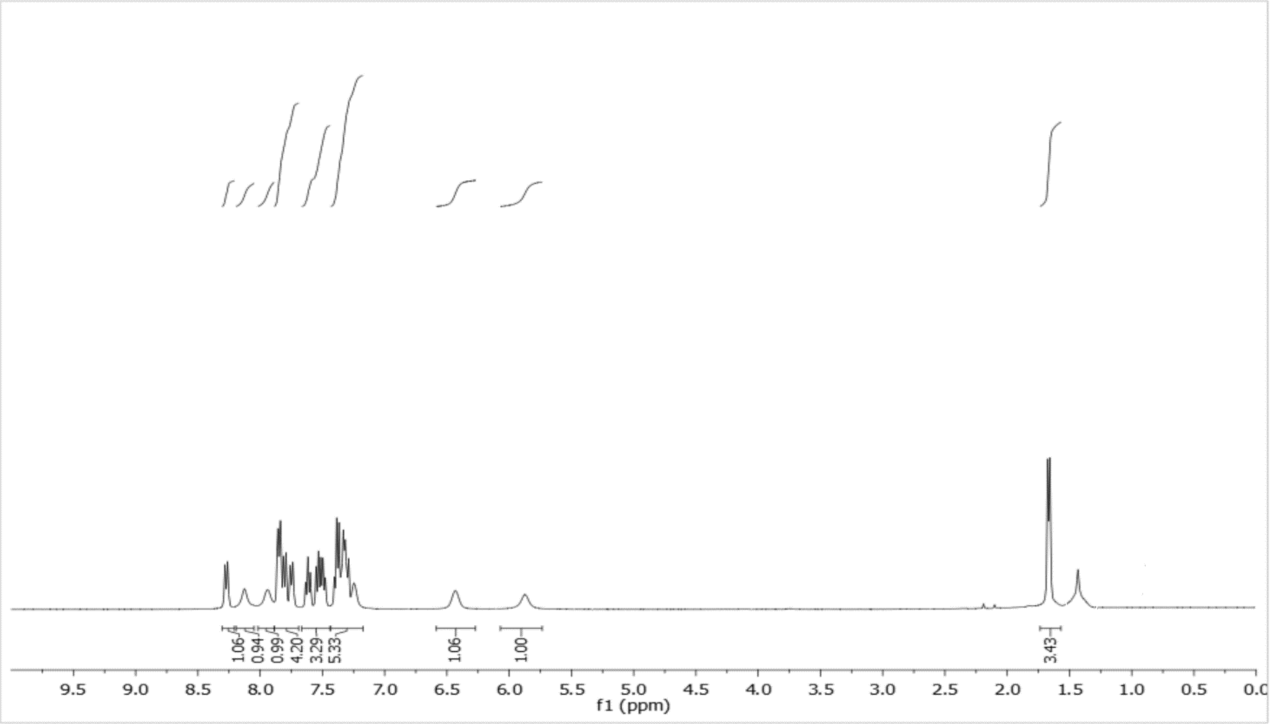

Figure S1. ^{1}H NMR spectrum of **S-1** in $CDCl_3$

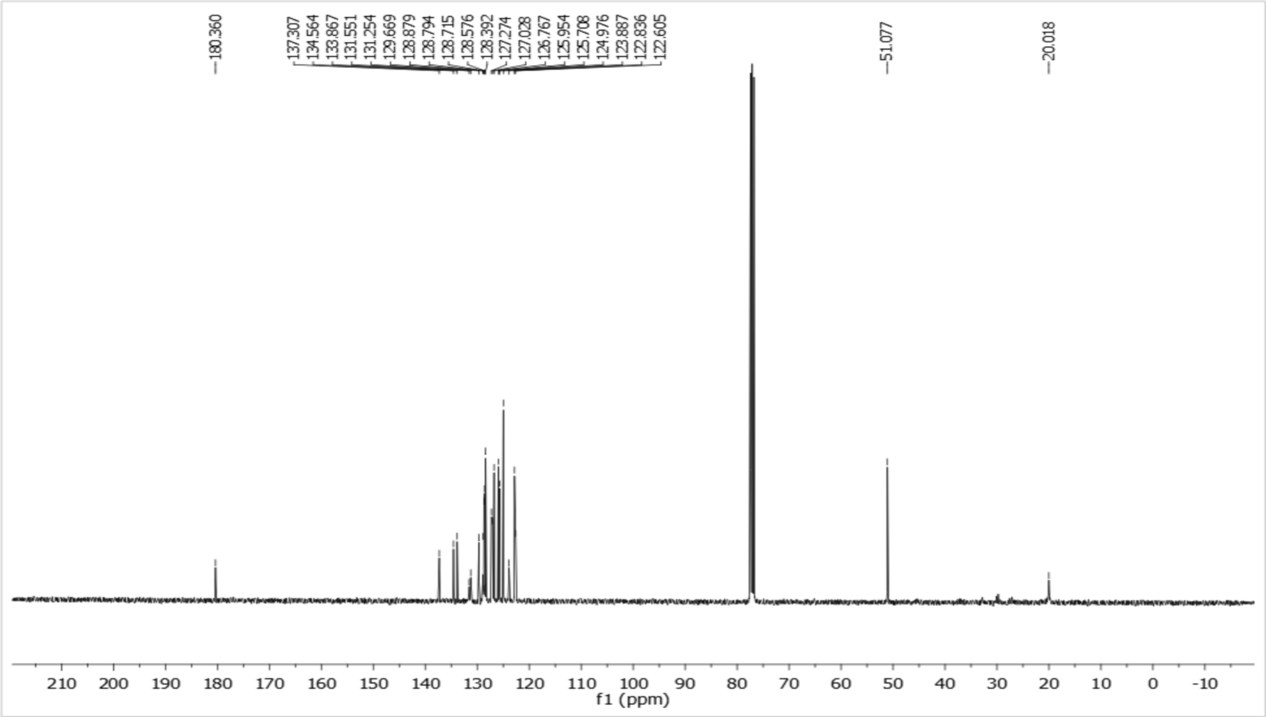

Figure S2. ^{13}C NMR spectrum of ***S*-1** in $CDCl_3$

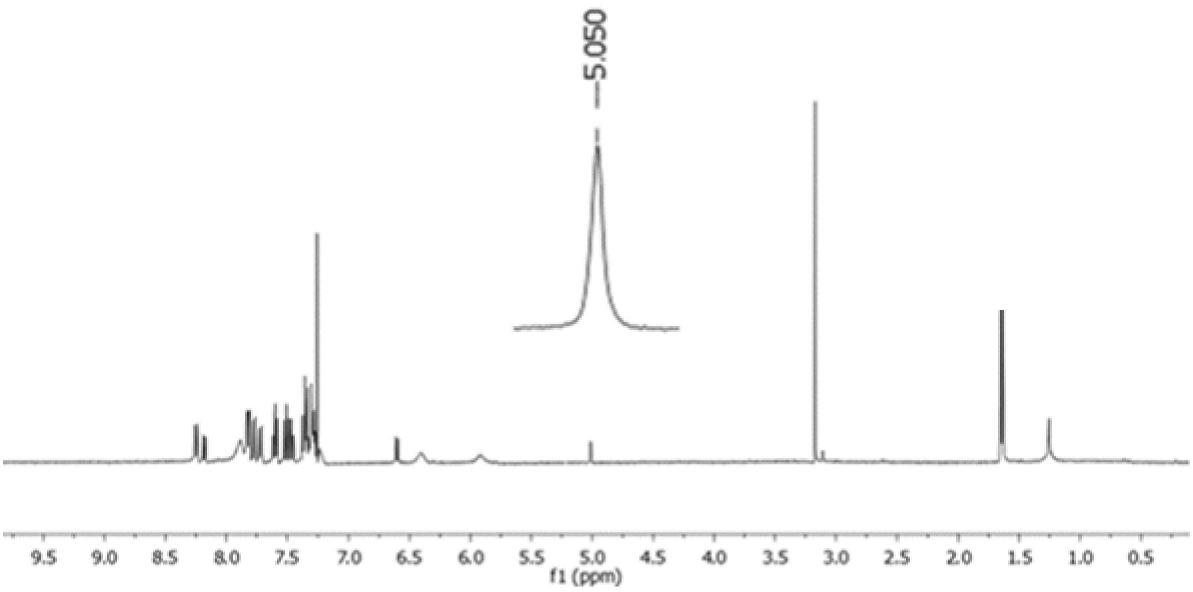

Figure S3. ^{1}H NMR spectrum of **1** in the presence of 1 equivalent of ***S*-1** and DMAP in $CDCl_3$

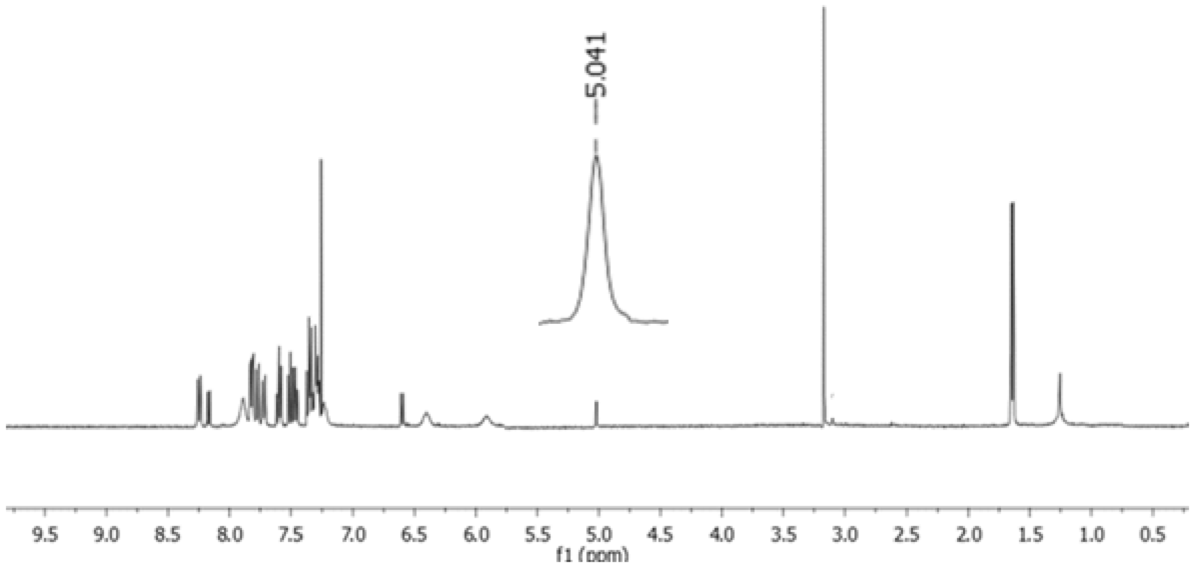

Figure S4. ^{1}H NMR spectrum **1** in the presence of 2 equivalents of ***S*-1** and DMAP in $CDCl_3$

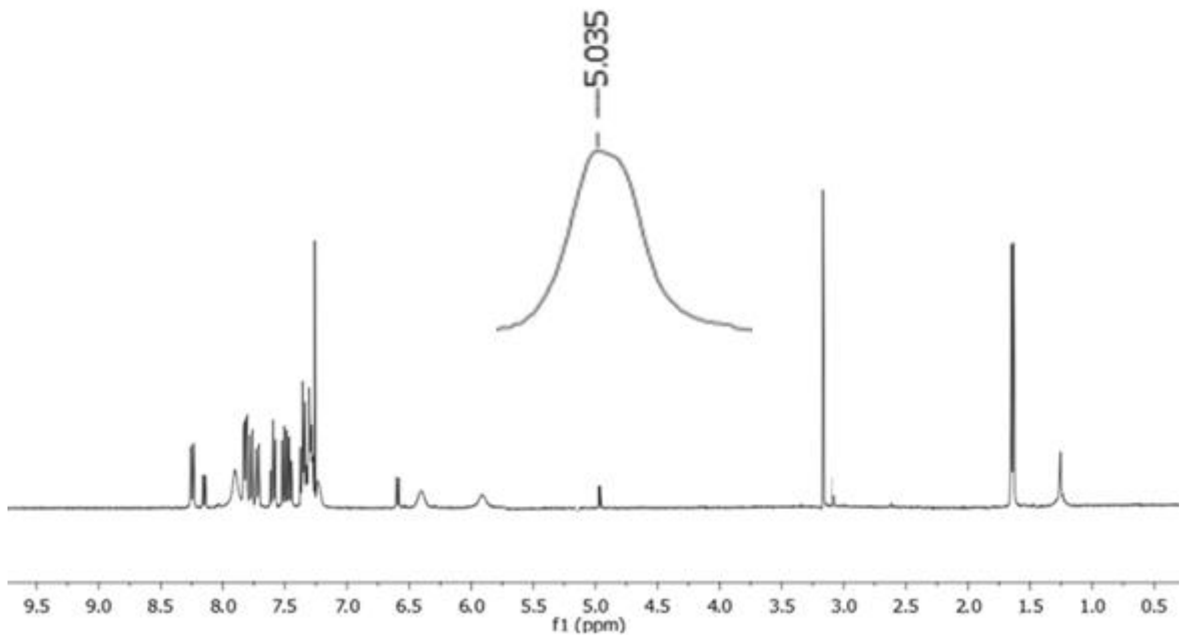

Figure S5. ^{1}H NMR spectrum **1** in the presence of 3 equivalents of ***S*-1** and DMAP in $CDCl_3$

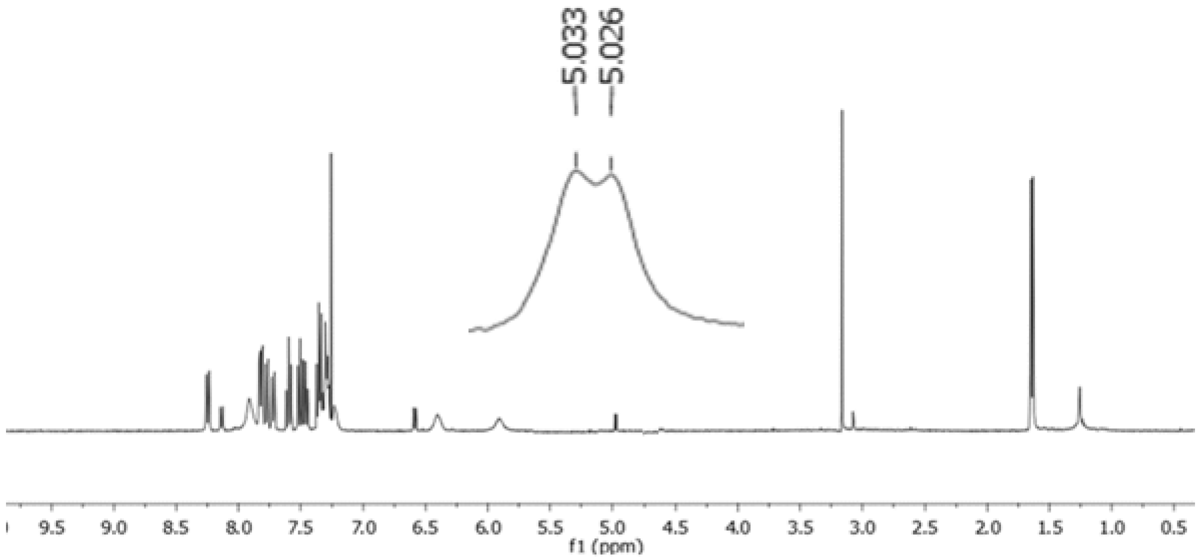

Figure S6. ^{1}H NMR spectrum of **1** in the presence of 4 equivalents of ***S*-1** and DMAP in $CDCl_3$

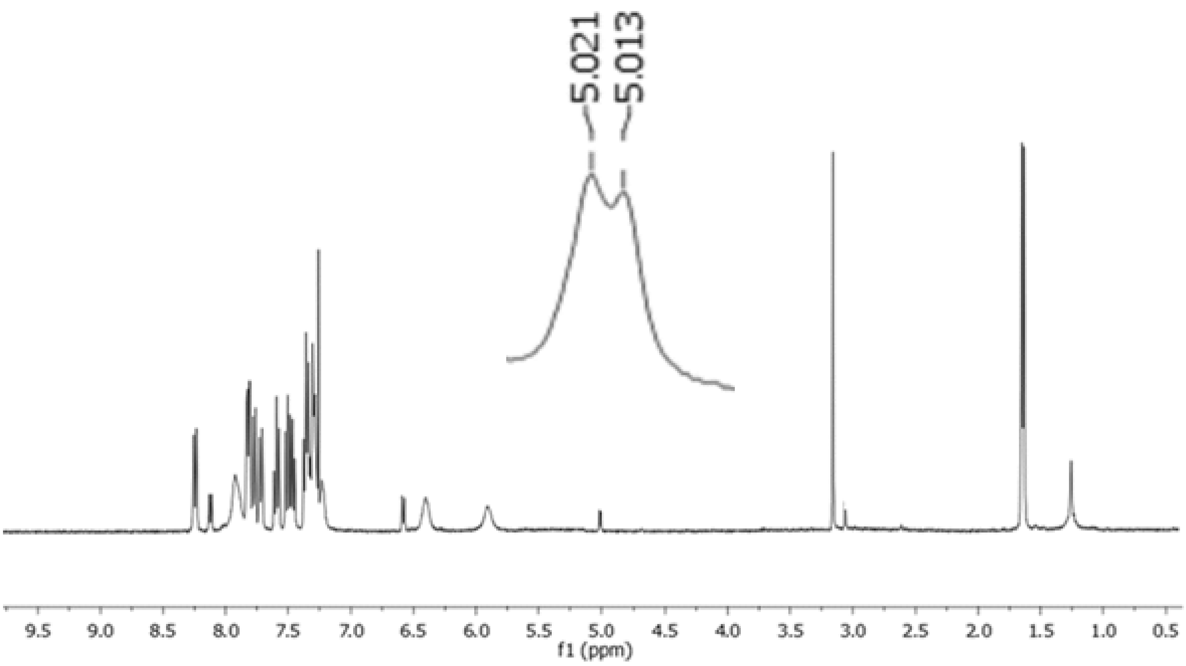

Figure S7. ^{1}H NMR spectrum of **1** in the presence of 5 equivalents of ***S*-1** and DMAP in $CDCl_3$

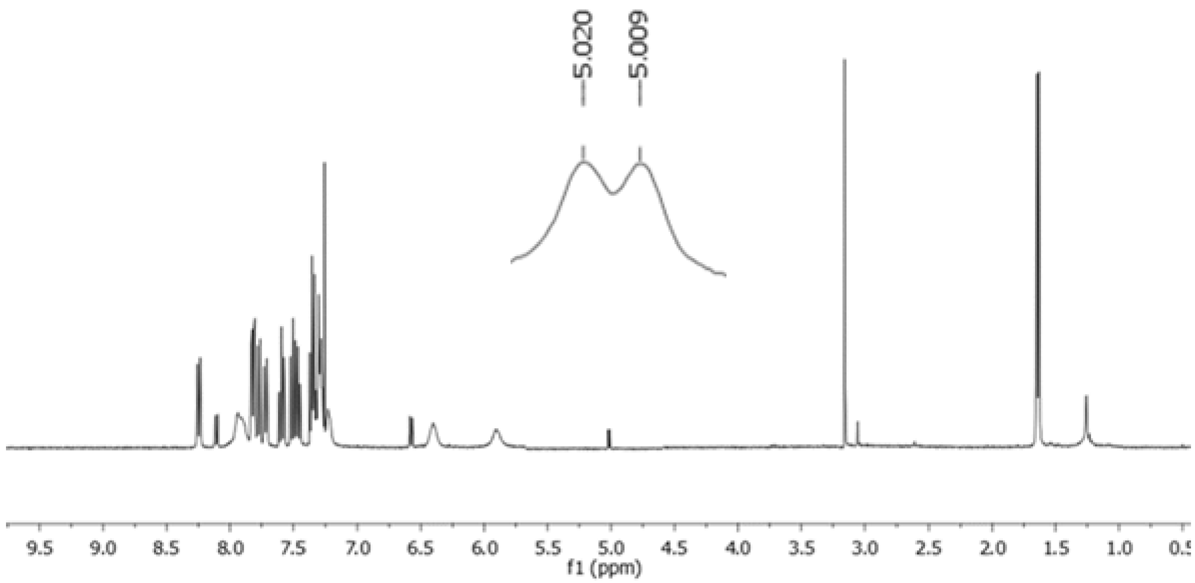

Figure S8. ^{1}H NMR spectrum of **1** in the presence of 6 equivalents of ***S*-1** and DMAP in $CDCl_3$

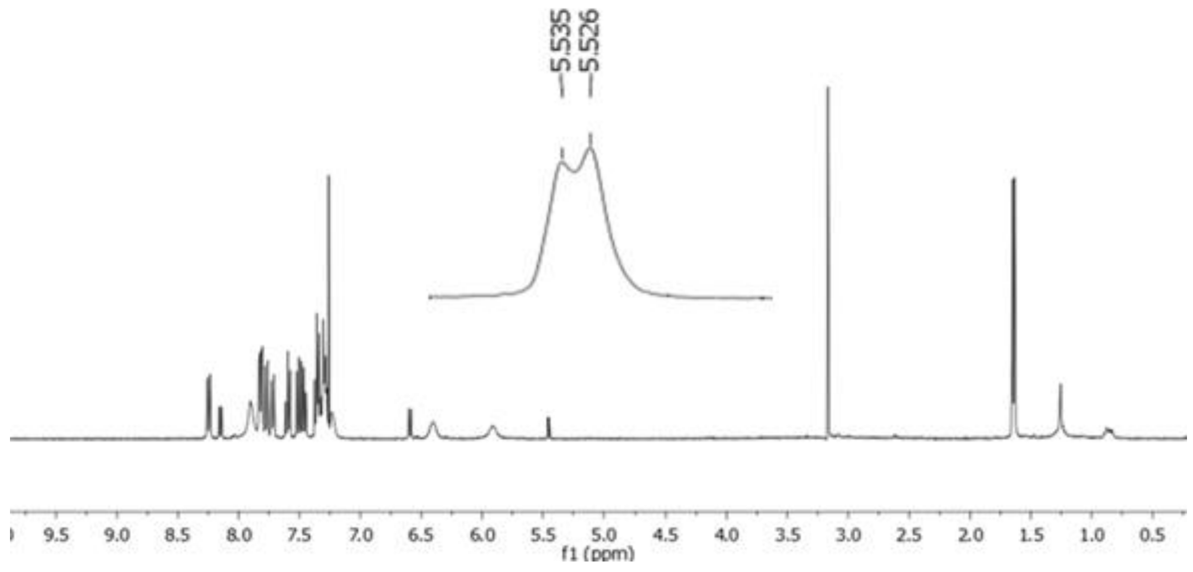

Figure S9. ^{1}H NMR spectrum of **2** in the presence of 3 equivalents of ***S*-1** and DMAP in $CDCl_3$

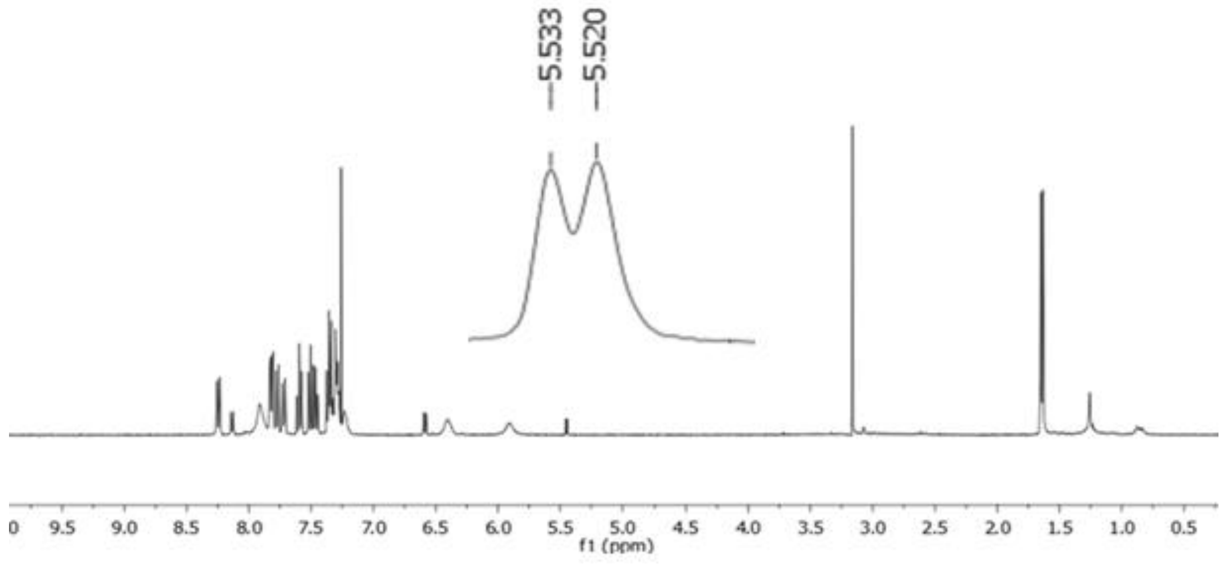

Figure S10. ^{1}H NMR spectrum of **2** in the presence of 4 equivalents of ***S*-1** and DMAP in $CDCl_3$

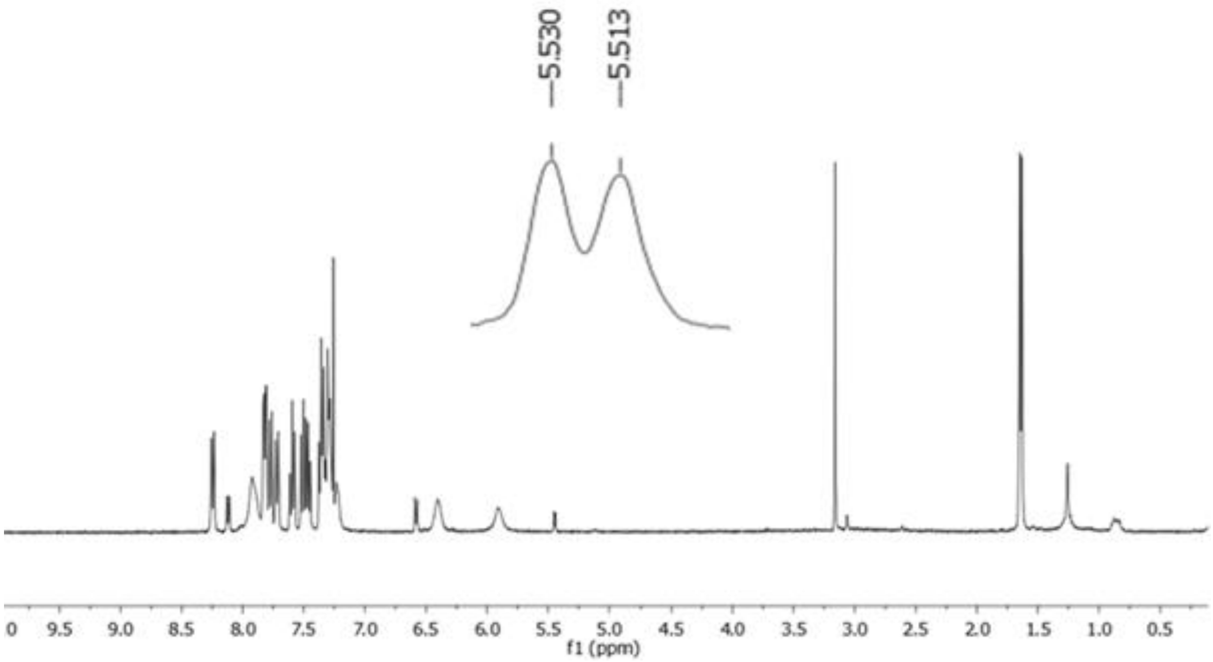

Figure S11. ^{1}H NMR spectrum of **2** in the presence of 5 equivalents of ***S*-1** and DMAP in $CDCl_3$

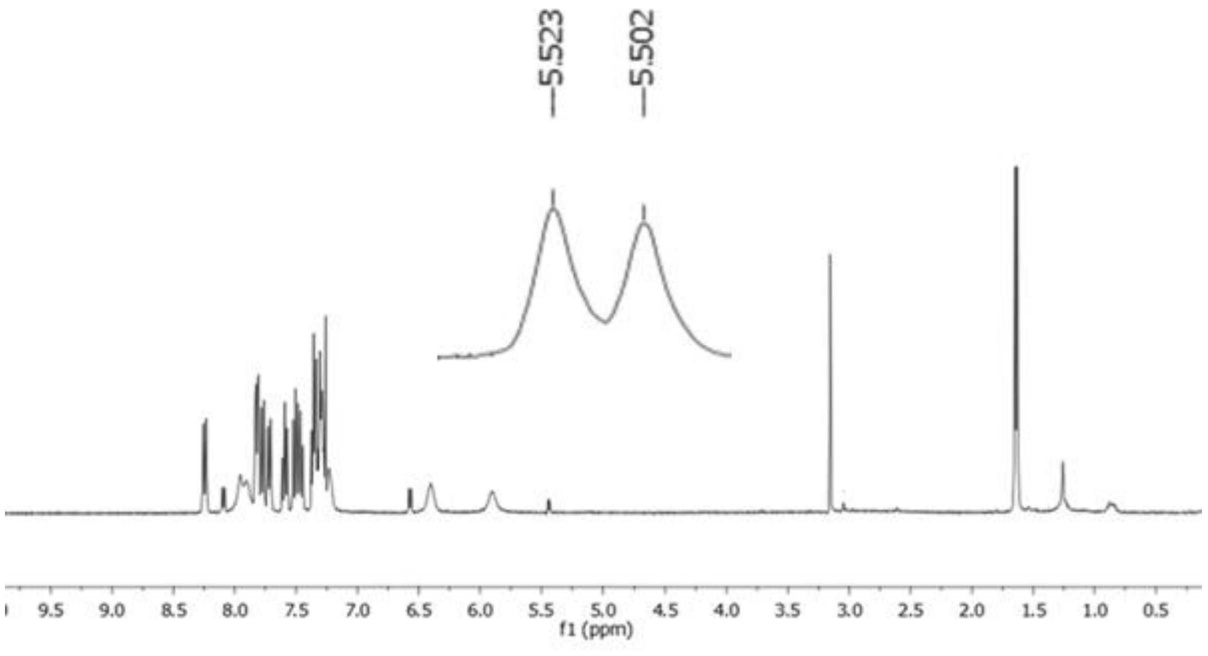

Figure S12. ^{1}H NMR spectrum of **2** in the presence of 6 equivalents of ***S*-1** and DMAP in $CDCl_3$

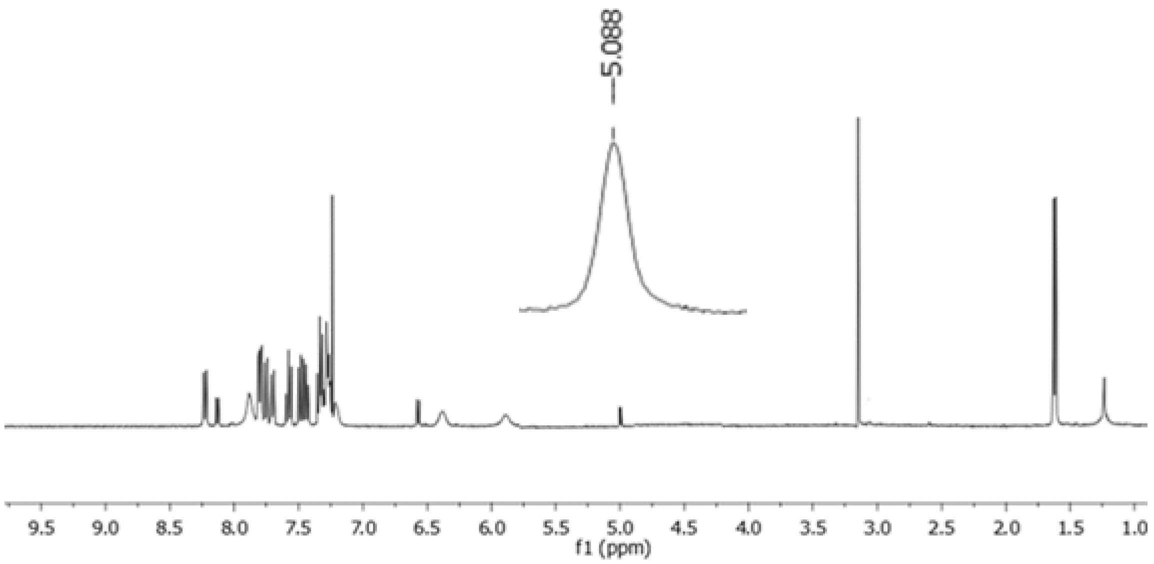

Figure S13. ^{1}H NMR spectrum of **3** in the presence of 3 equivalents of ***S*-1** and DMAP in $CDCl_3$

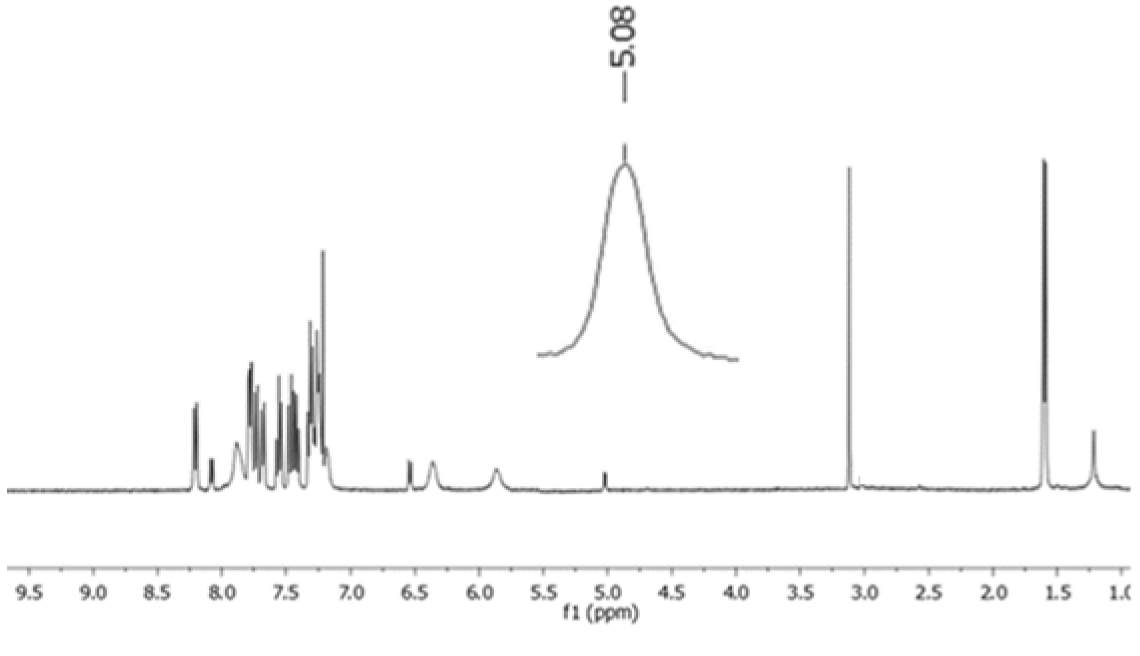

Figure S14. ^{1}H NMR spectrum of **3** in the presence of 4 equivalents of ***S*-1** and DMAP in $CDCl_3$

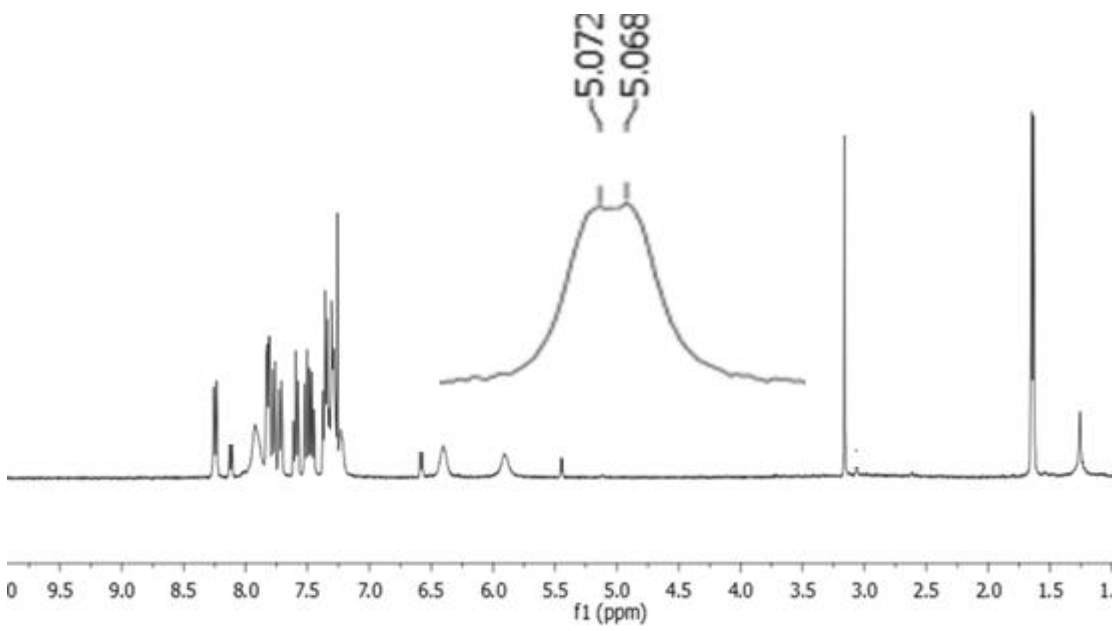

Figure S15. ^{1}H NMR spectrum of **3** in the presence of 5 equivalents of ***S*-1** and DMAP in $CDCl_3$

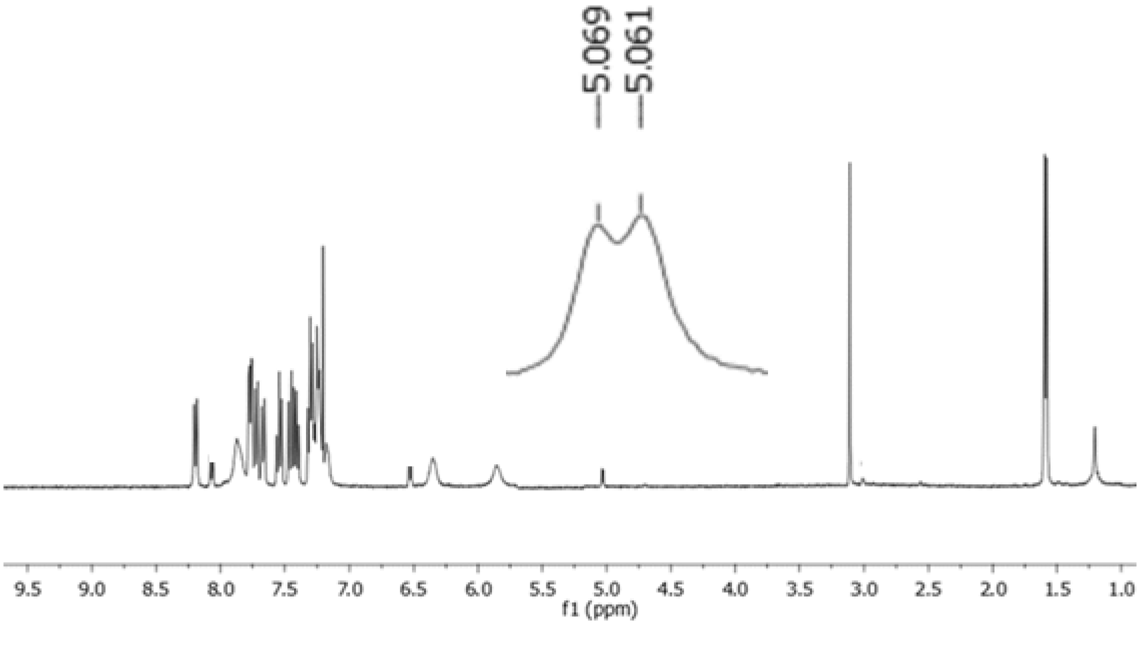

Figure S16. ^{1}H NMR spectrum of **3** in the presence of 6 equivalents of ***S*-1** and DMAP in $CDCl_3$

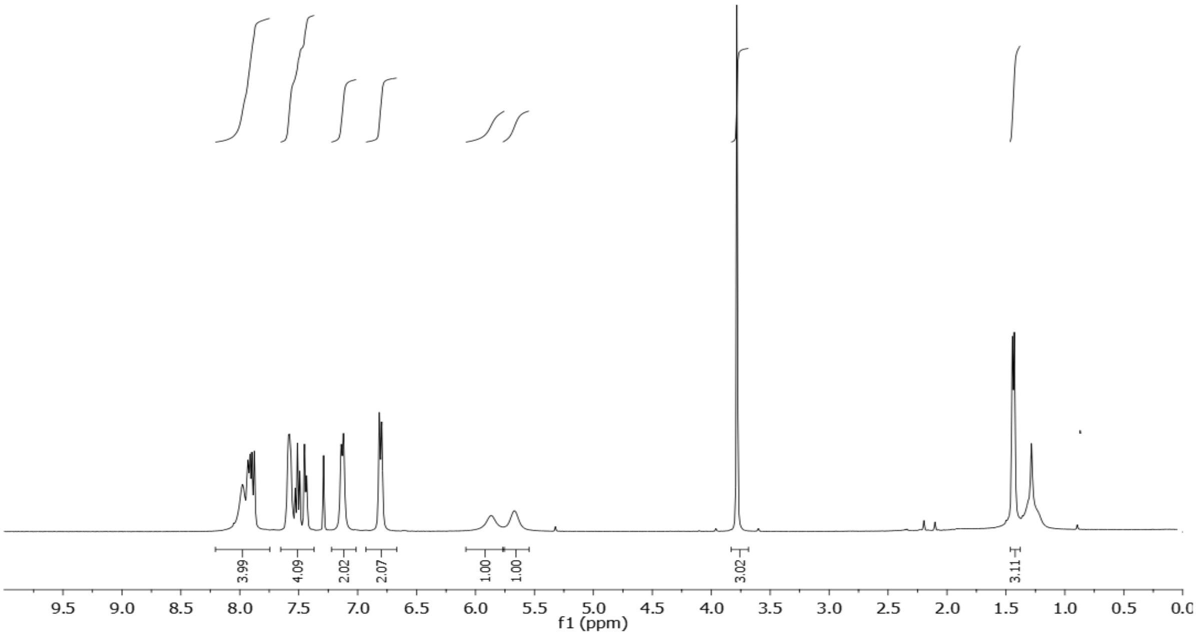

Figure S17. ^{1}H NMR spectrum of ***S*-2** in $CDCl_3$

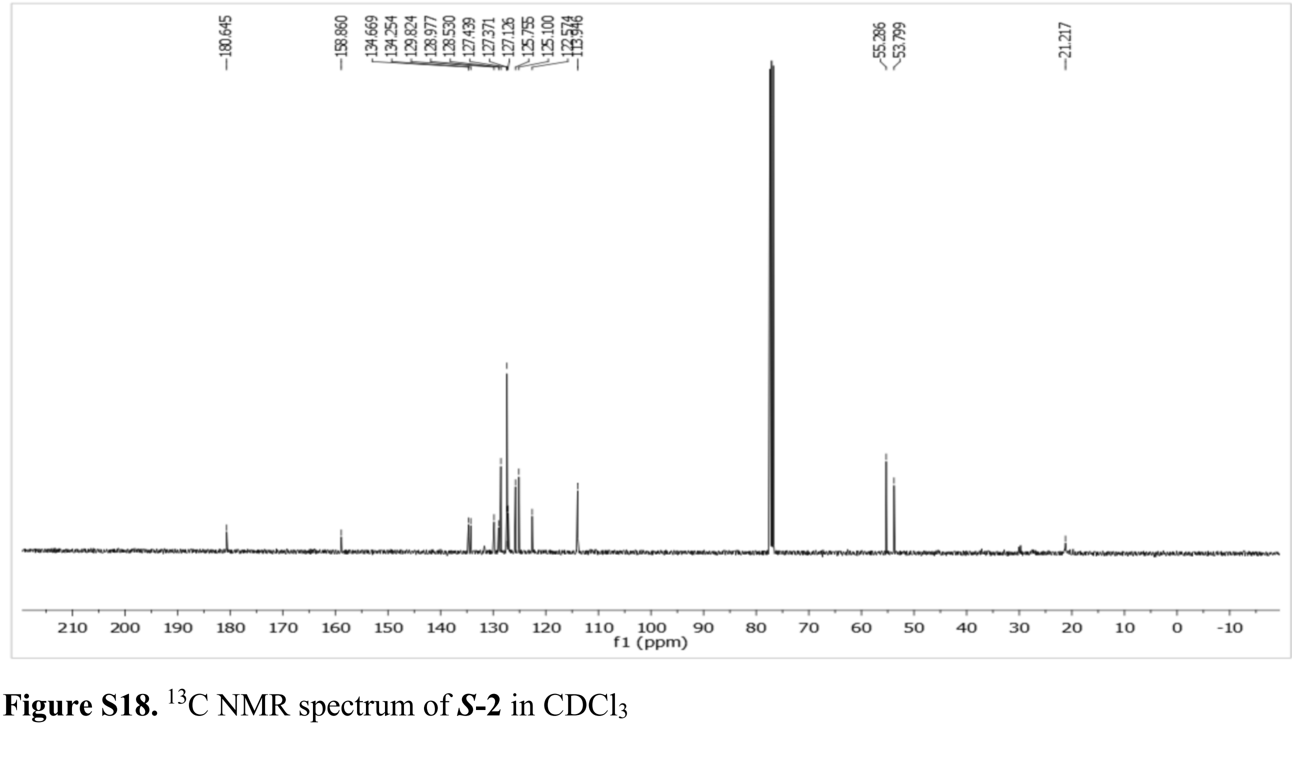

Figure S18. ^{13}C NMR spectrum of ***S*-2** in $CDCl_3$

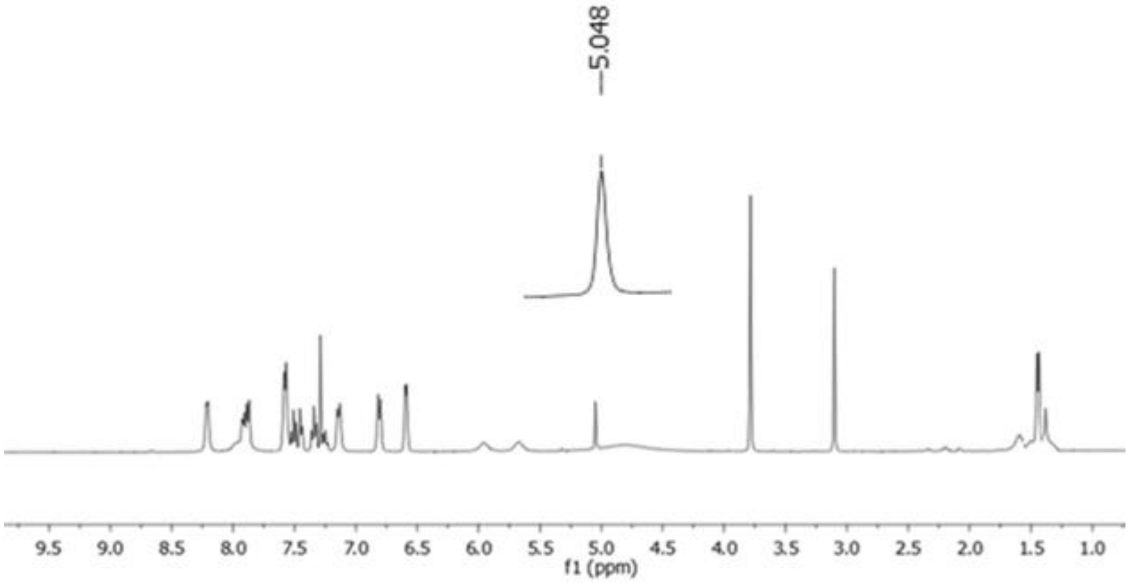

Figure S19. ^{1}H NMR spectrum of **1** in the presence of 3 equivalents of ***S*-2** and DMAP in $CDCl_3$

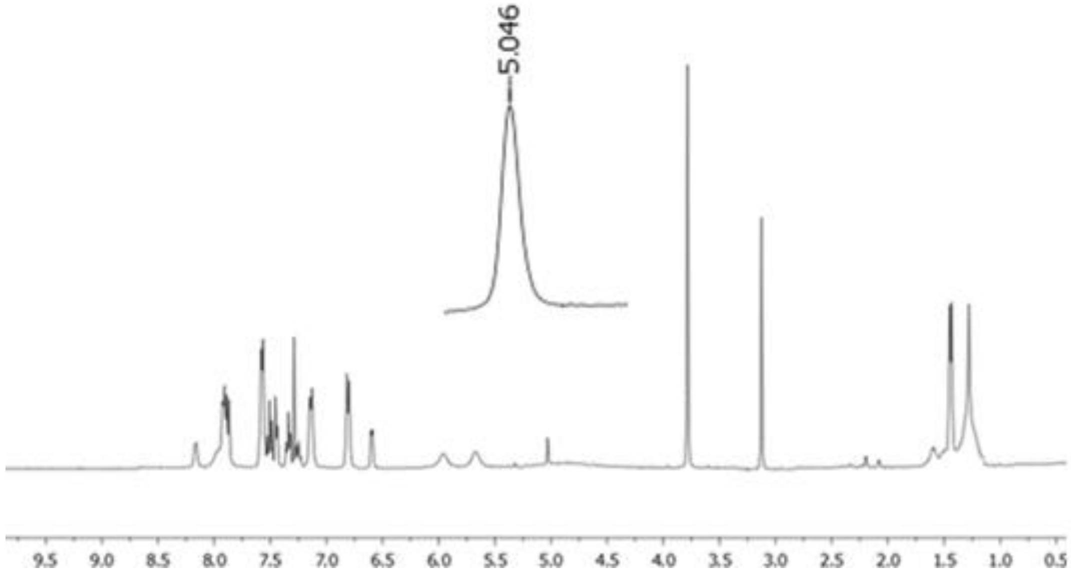

Figure S20. ^{1}H NMR spectrum of **1** in the presence of 4 equivalents of ***S*-2** and DMAP in $CDCl_3$

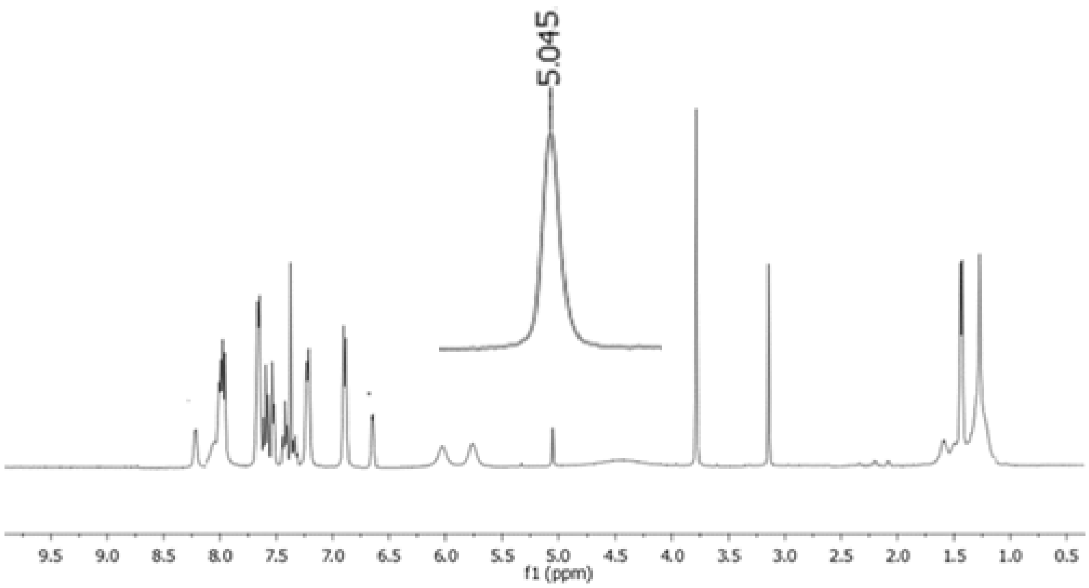

Figure S21. ^{1}H NMR spectrum of **1** in the presence of 5 equivalents of ***S*-2** and DMAP in $CDCl_3$

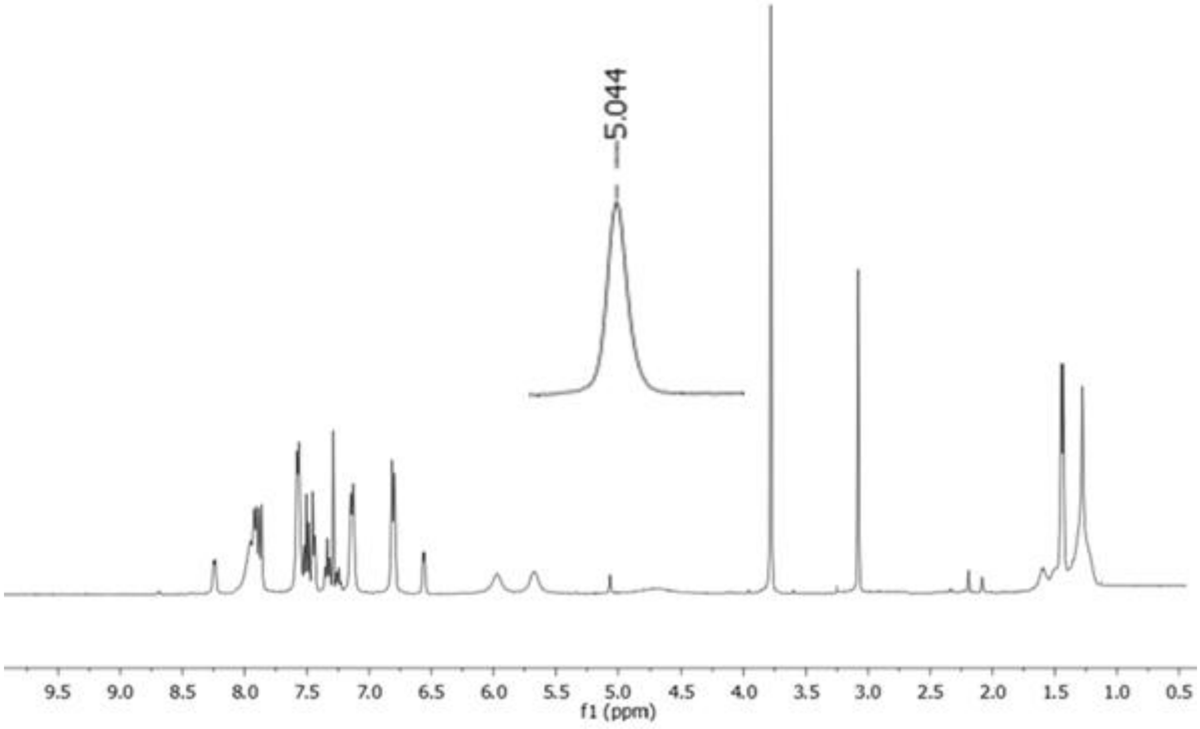

Figure S22. ^{1}H spectrum of **1** in the presence of 6 equivalents of ***S*-2** and DMAP in $CDCl_3$

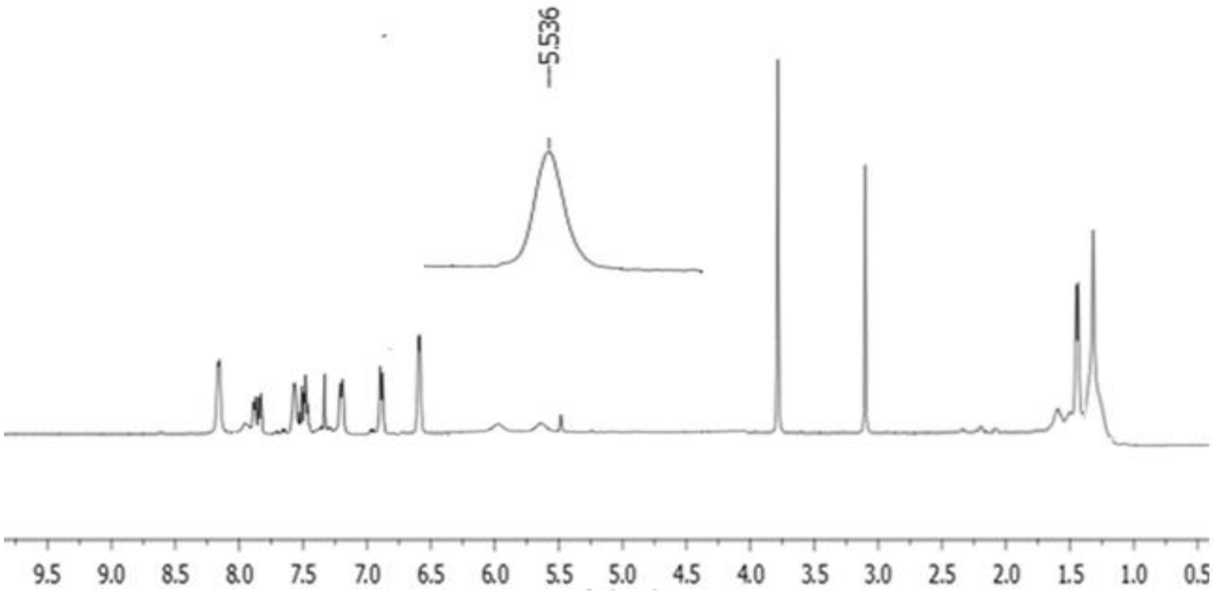

Figure S23. ^{1}H NMR spectrum of **2** in the presence of 3 equivalents of ***S*-2** and DMAP in $CDCl_3$

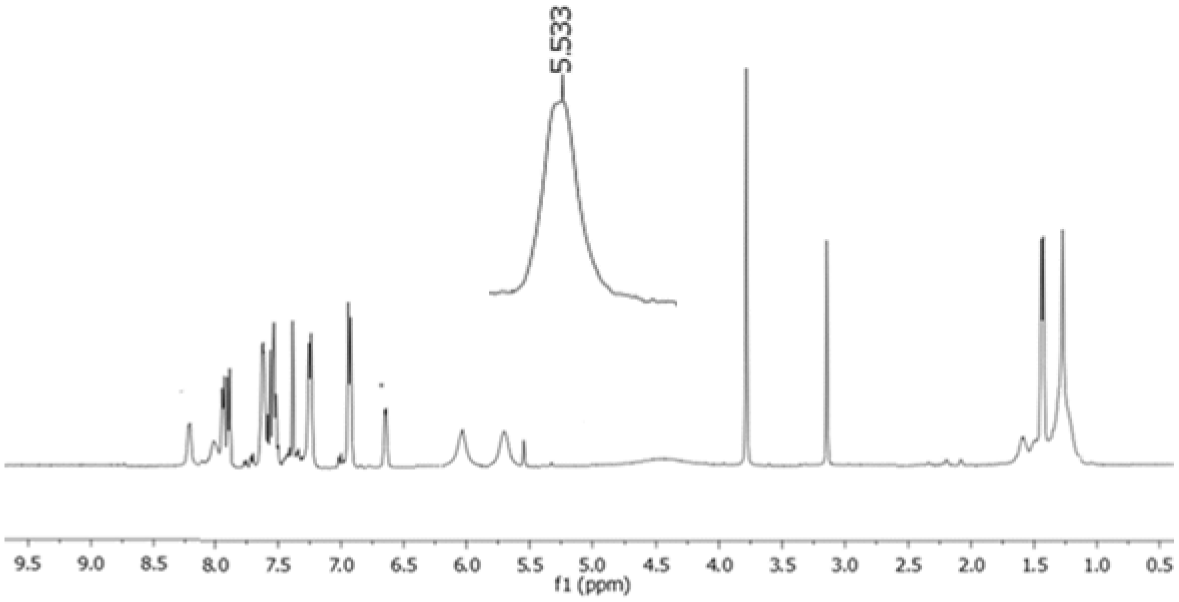

Figure S24. ^{1}H NMR spectrum of **2** in the presence of 4 equivalents of ***S*-2** and DMAP in $CDCl_3$

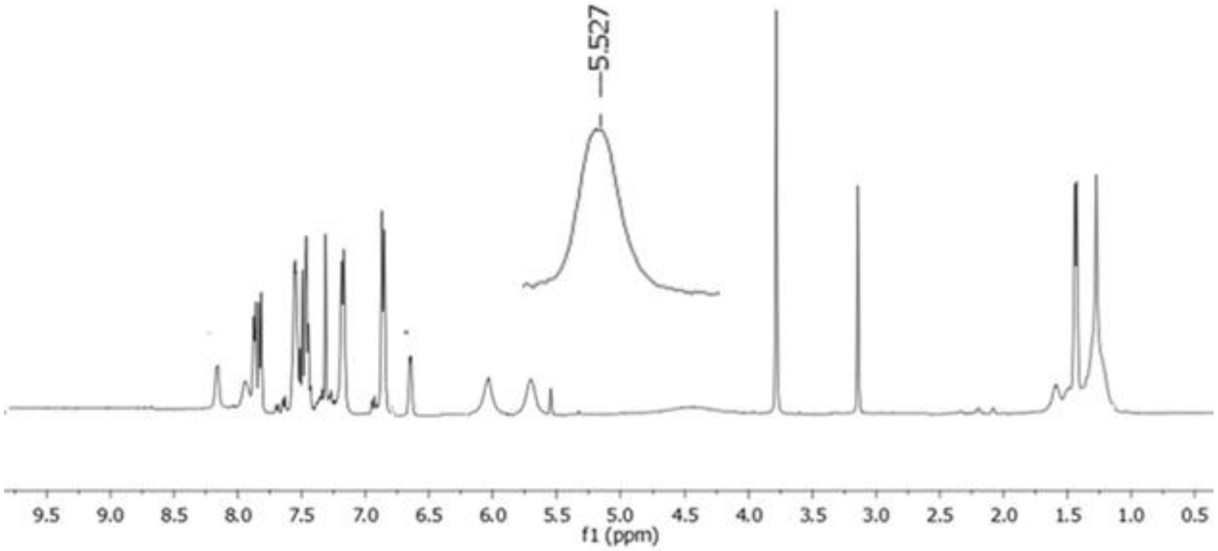

Figure S25. ^{1}H NMR spectrum of **2** in the presence of 5 equivalents of ***S*-2** and DMAP in $CDCl_3$

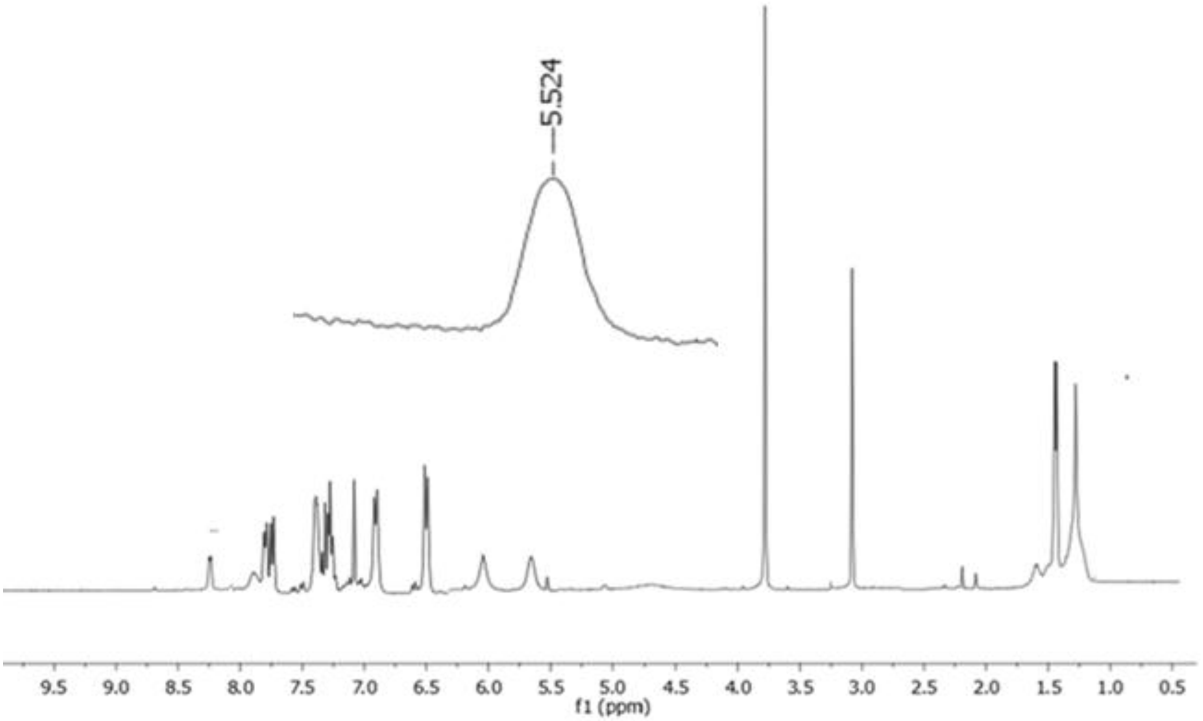

Figure S26. ^{1}H NMR spectrum of **2** in the presence of 6 equivalents of ***S*-2** and DMAP in $CDCl_3$

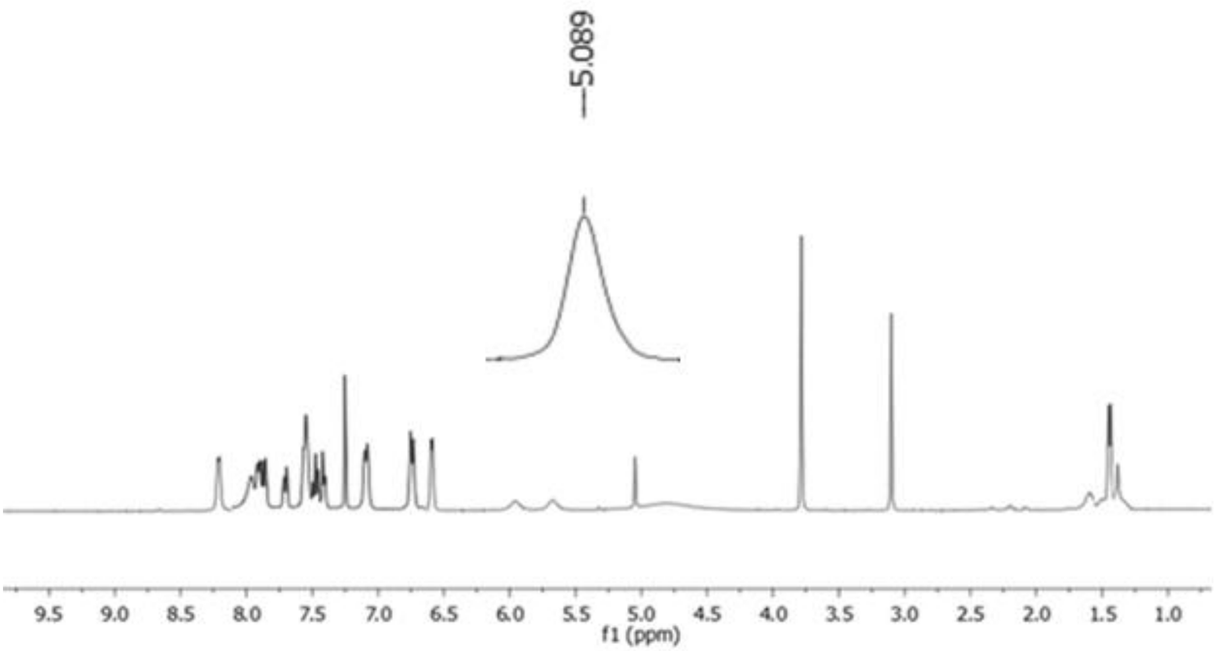

Figure S27. ^{1}H NMR spectrum of **3** in the presence of 3 equivalents of ***S*-2** and DMAP in $CDCl_3$

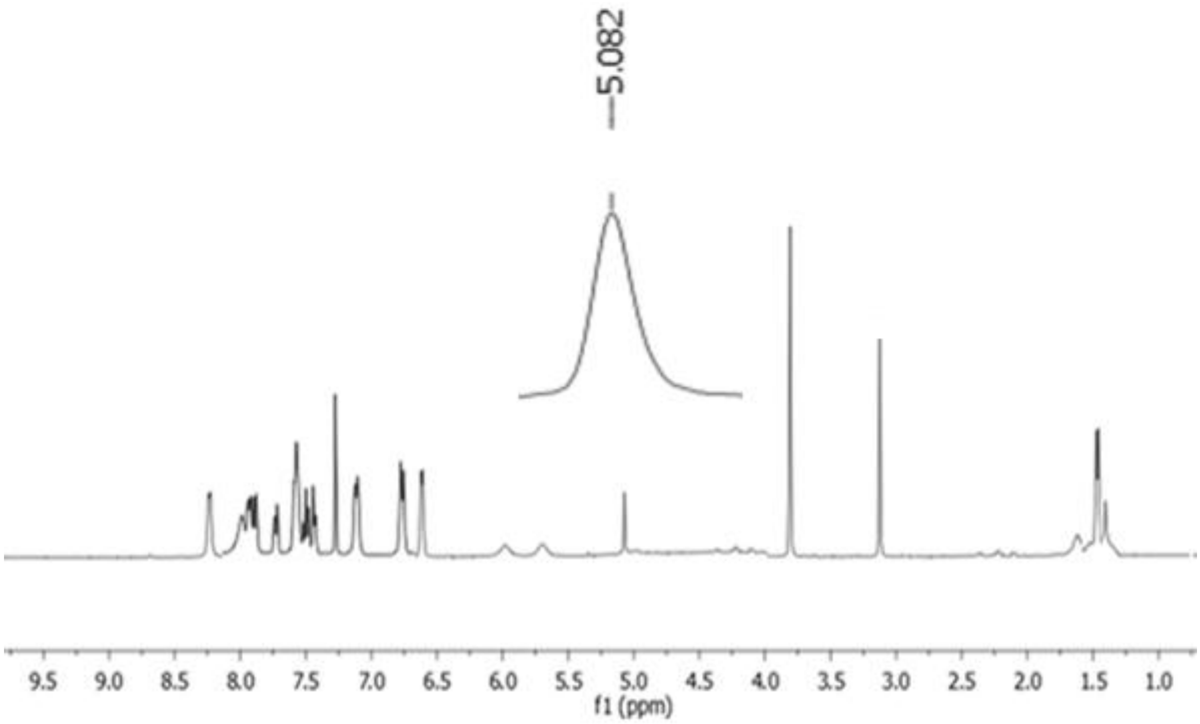

Figure S28. ^{1}H NMR spectrum of **3** in the presence of 4 equivalents of ***S*-2** and DMAP in $CDCl_3$

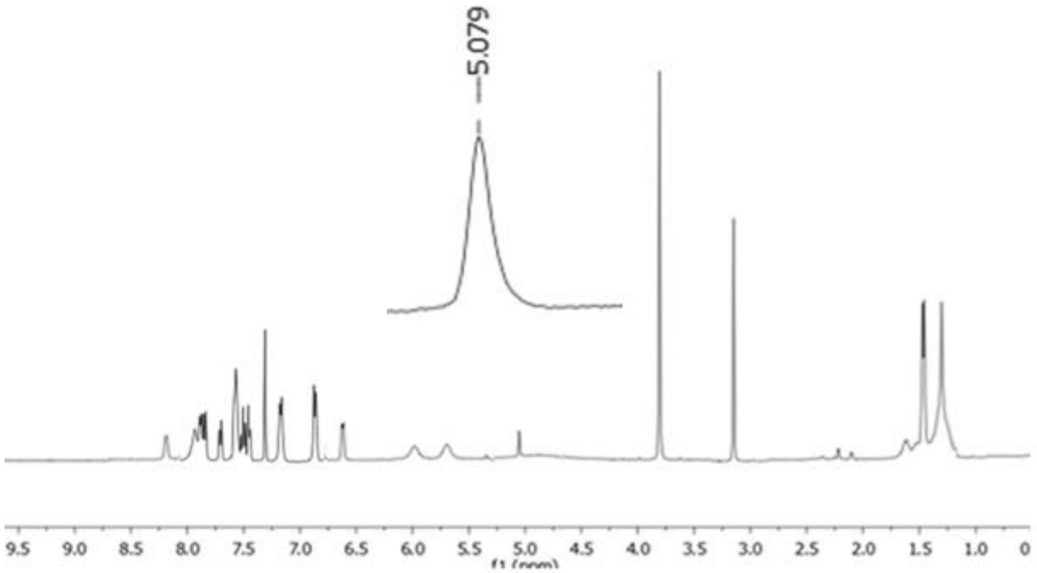

Figure S29. ^{1}H NMR spectrum of **3** in the presence of 5 equivalents of ***S*-2** and DMAP in $CDCl_3$

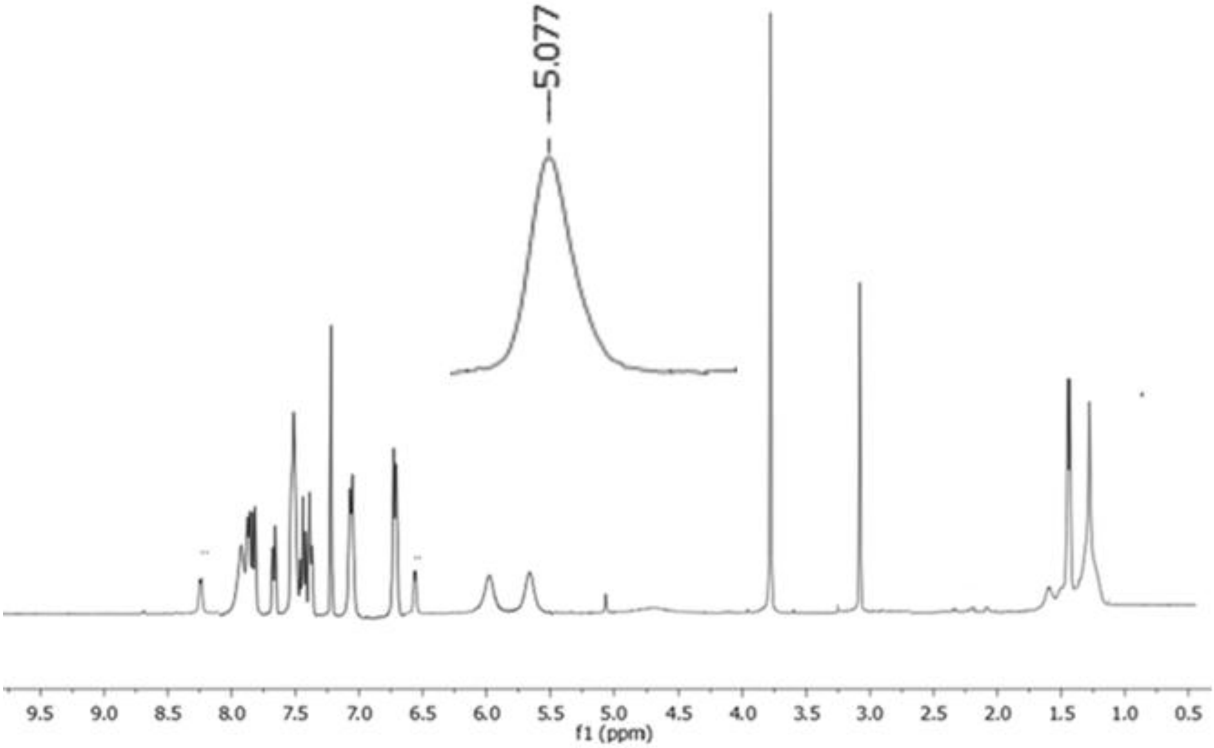

Figure S30. ^{1}H NMR spectrum of **3** in the presence of 6 equivalents of ***S*-2** and DMAP in $CDCl_3$

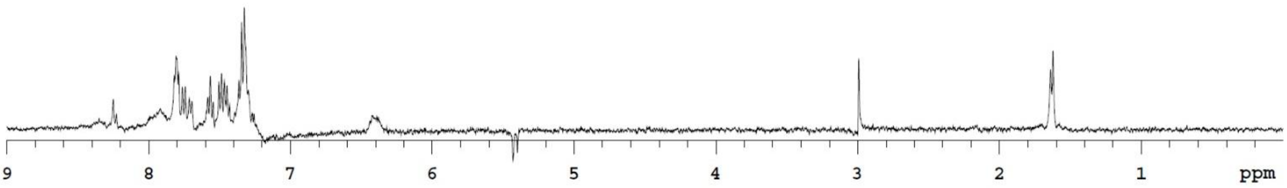

Figure S31. 1D-ROESY (400 MHz, $CDCl_3$, 298 K, mixing time 500 ms) spectrum of complex formed between S-1 and substrate 2

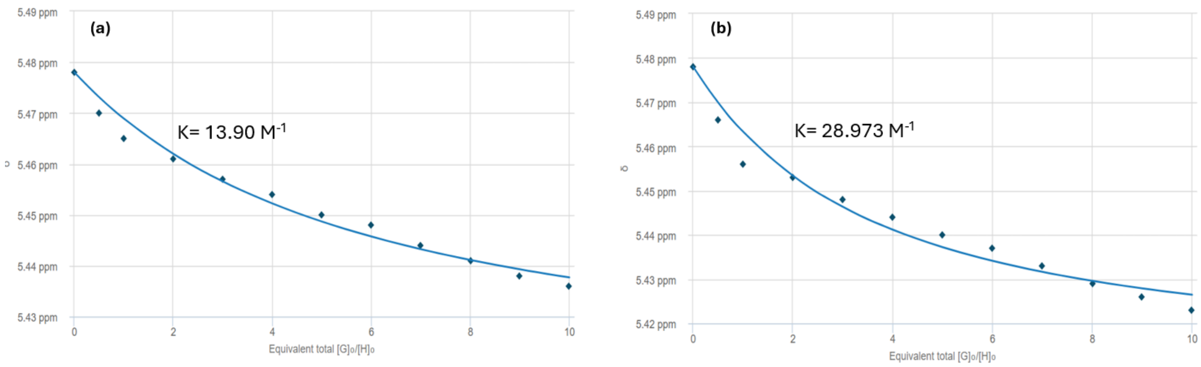

Figure S32. Association constants calculated for a) R enantiomer and b) S enantiomer of 2 with S-1 in the presence of DMAP.

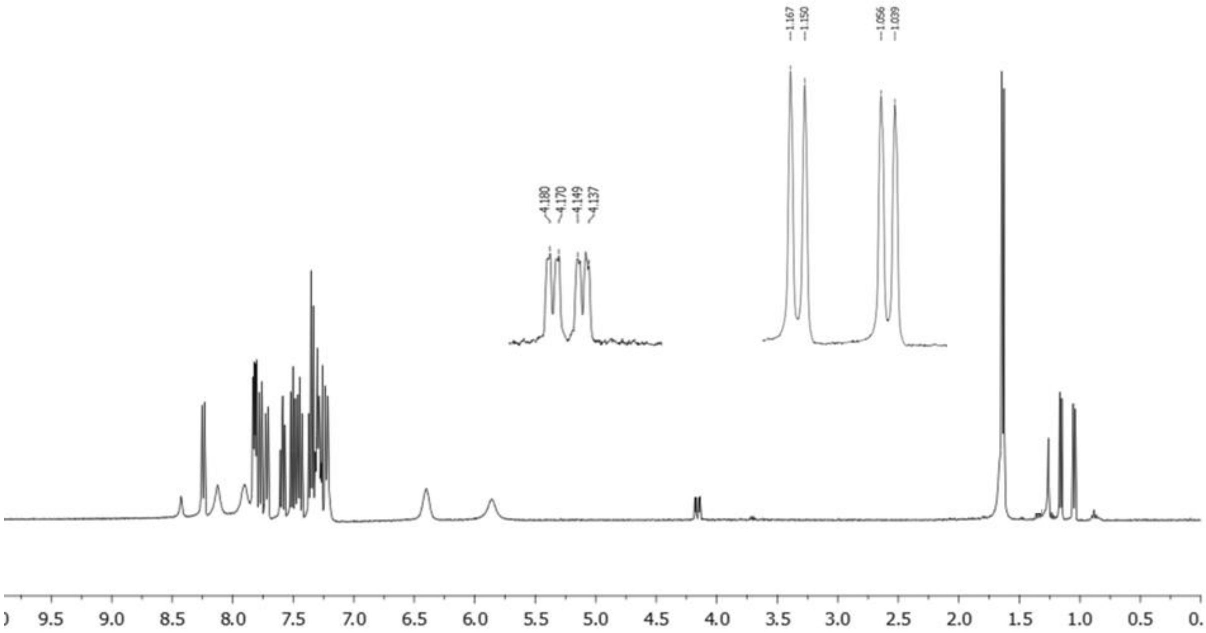

Figure S33. ^{1}H NMR spectrum of **4** in the presence of 6 equivalents of ***S*-1** in $CDCl_3$.

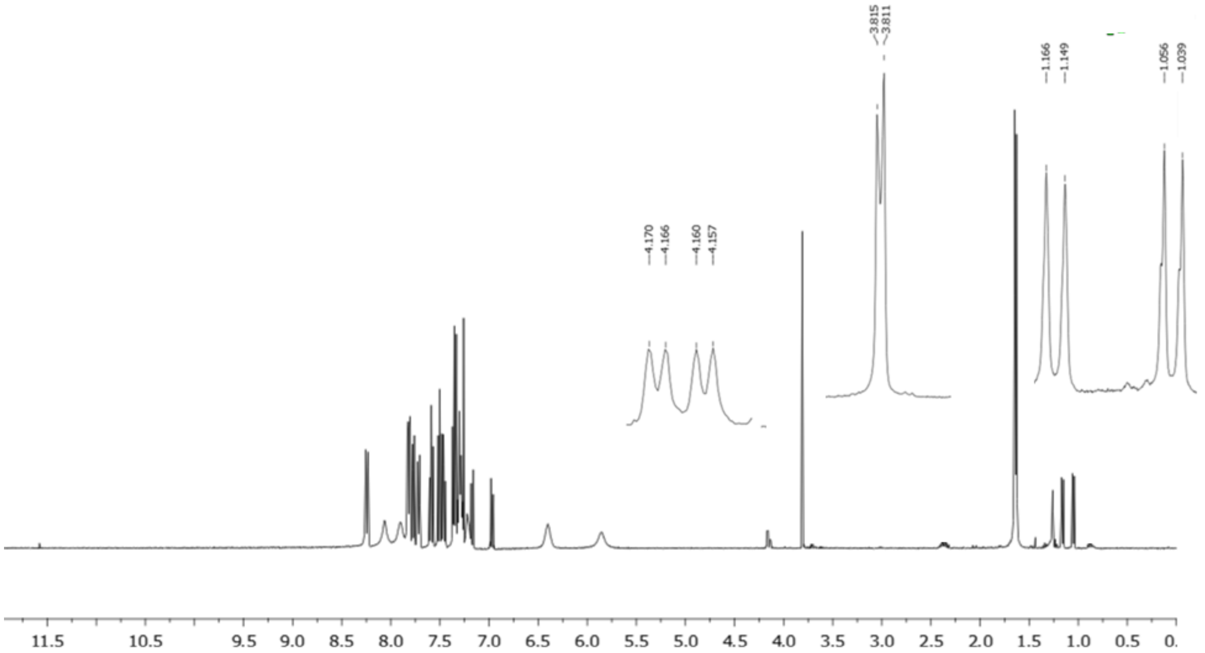

Figure S34. ^{1}H NMR spectrum of **5** in the presence of 6 equivalents of ***S*-1** in $CDCl_3$.
